# The Indian regulatory framework and the surge of unproven stem cell therapies—a call for diagnosis

**DOI:** 10.1093/jlb/lsaf027

**Published:** 2025-11-20

**Authors:** Vaishnav M

**Affiliations:** National University of Advanced Legal Studies, Kochi 683 503, Kerala, India

**Keywords:** stem cell regulation, unproven stem cell therapies, stem cell tourism, Indian regulatory framework

## Abstract

Stem cell science lies at the heart of regenerative medicine; it is believed to have the potential to address several otherwise incurable diseases. However, even though stem cell interventions are at the experimental stage, they are advertised as a panacea for various human maladies. India, like other jurisdictions across the globe, has witnessed the mushrooming of ‘experts’ and ‘clinics’ selling (unproven) stem cell therapies to vulnerable and desperate patients. The unscrupulous activities in the stem cell industry endanger patients and hinder legitimate scientific progress. In light of the crisis of unproven stem cell therapies, I seek to comprehensively study the Indian regulatory framework on stem cells. The article identifies multiple issues in the framework—the inherent ambiguity in the governing laws, the unscientific functioning of the regulatory institutions, the poor implementation of the existing laws, and the seemingly consequent rise of India as an unregulated stem cell tourism hub. The suggested reforms broadly include streamlined funding, consolidated clinical regulation, and public engagement. The policymakers shall consider redefining their playbook to facilitate a conducive atmosphere for the growth of stem cell science.

## I. INTRODUCTION

With the advent of the 21st century, stem cell research has acquired considerable prominence and many countries across the globe have actively taken up this project.^1^ Starting from restoration of vision to curing diabetes, stem cell–based clinical trials have hit the headlines multiple times in the past months.^2^

A stem cell is a type of cell that can differentiate itself and regenerate into a large number of new cells.^3^ Stem cell science is witnessing ground-breaking academic inquiries,^4^ and it appears to offer promising therapeutic applications, thanks to its self-renewal capabilities.^5^ At the global level, the International Society for Stem Cell Research promotes ethical research in the field and issues guidelines and standards for stem cell clinical trials.^6^

While we have these international guidelines, the degree and nature of regulations vary widely from one country to another. For example, while stem cell research is more tightly regulated in India through licensing and approval procedures, the process is more relaxed in some countries like the USA.^7^ Today, India is seen as one among the promising destinations for cell therapy with further potential for growth.^8^ This prompts a review of the readiness of regulatory practice in India.

Indian law categorizes stem cell derivatives as a ‘new drug’ for the purposes of clinical applications.^9^ As a result, regulation of its research falls squarely under the broader framework of drug regulation. The incorporation of stem cells within the drug regulatory framework has been a slow process—it was only in 2018 that a proposal was made to categorize stem cells as drug and thereby bring it under the regulatory scanner.^10^ However, before this proposal materialized, the New Drugs and Clinical Trial Rules, 2019 was released, which introduced the category of ‘new drugs’, bringing in ‘cell- or stem cell–derived product’ in its ambit.^11^

Parallel to these legal developments, we have witnessed the rise of several government-funded research projects related to stem cells in the past decade.^12^ Unlike its European counterparts, India has adopted a relatively liberal stance towards embryonic stem cell research.^13^ It has witnessed an appreciable growth in stem cell research and clinical trials.^14^ Albeit, it appears that at a global level, the quality of India’s research output continues to be sub-par.^15^ It can be, among others, attributed to the absence of a robust regulatory framework, a problem that is the focal point of my analysis.^16^

The concern here is not just about protecting the research environment. The safety of patients who fall prey to the tall promises of unscrupulous actors in the stem cell industry is an equally grave, if not graver, concern.^17^ Considering the alarming nature of the matter, the paper calls for policy intervention to protect the interests of the patients and consumers.

The primary agenda of this paper is to advance a set of reforms India can adopt to optimize its regulatory practices. The first part of the article sets out the existing regulatory framework characterized by overlapping laws and guidelines and overburdened regulators. While the existing literature highlights several issues in the regulatory framework, the discussion has been quite generic from a legal scholarship viewpoint. Hence, this part seeks to firstly, take up a legal analysis of the laws underpinning the framework, and secondly, to link the specific legal inconsistencies to the practical effect of regulatory failure.

I engage with various primary documents to explain the regulation of different aspects of stem cell science—namely, clinical trials, therapies, use of human embryonic stem cells (hESCs), stem cell banking, and advertisements. To make up for the gap in the information, and to confirm some of the inferences, the responses received to the ‘Right to Information’ queries filed by the author has been relied upon.

The second part of the article discusses the systemic defects plaguing the regulatory mechanism. These systemic defects deter quality research and facilitate the proliferation of clinics offering unproven therapies under false and unscientific promises.

The reforms suggested in Section IV broadly cover the areas of funding, clinical regulation, and public engagement. While acknowledging the inherent difficulties, the article proposes enactment of a consolidating law to harmonize the regulation of stem cell research. Yet, a clear and fine piece of law alone might not suffice. Through this article, I re-emphasize the long-standing demand for restructuring the institutional framework involving the Central Drugs Standards Control Organization (CDSCO) and the State Drugs Regulatory Authorities (SDRAs) to ensure better coordination and uniformity in the practices. Further, it is necessary to facilitate structured state funding of research to ensure a holistic growth of stem cell science in India. It is important to allocate state funds to support projects in the interest of the public that are otherwise ignored by profit-driven private laboratories and organizations. As a final measure, I highlight the importance of sensitizing the public and empathetically engaging with the patients through support groups, to prevent exploitation and to support those in distress.

While the existing literature provides principled discussion on the issues foundational to the regulatory failure,^18^ it does not extensively and conclusively discuss the specific legal and policy changes that can be brought in a country like India. I attempt to highlight particular measures for reform, starting from statutory measures to direct public engagement. And in this process, I also attempt to close the chasm that exists between one part of literature that broadly calls for positive legal reforms^19^ and the other part highlighting the limitations of such measures.^20^

While many social scientists have highlighted the inadequacy of a new law, the article tries to convey that a clear, consolidated statute is necessary in the Indian scenario. But such a law and the surrounding institutional mechanism must account for the political, economic, and social forces. This inquiry aims to lay a groundwork for the legislature and the regulators in tackling the issue and possibly act as a legislative exemplar for other countries in a similar bio-legal context.

## II. THE INDIAN REGULATORY FRAMEWORK ON STEM CELLS

Indian policymakers’ efforts to monitor stem cell research began at the dawn of the 21st century.^21^ In 2001, the Department of Biotechnology (DBT) issued guidelines in response to the rising number of instances of misuse of stem cell science for unapproved clinical applications.^22^ Later, in 2002, the Indian Council of Medical Research (ICMR) announced a policy in favor of stem cell research and therapeutic application.^23^ It was only in 2007 that both the DBT and the ICMR, the two principal policy-formulating institutions,^24^ came together to issue national-level guidelines.^25^ Since then, there have been quite a few regulatory developments like the establishment of the Cell Biology Based Therapeutic Drug Evaluation Committee (CBBTDEC) within the CDSCO in 2010, and the enactment of the specific clinical trial rules in 2019.^26^

Currently, stem cells are regulated through the drug regulatory framework, and hence, it comes under the ambit of Drugs & Cosmetics Act, 1940 (D&C Act).^27^ However, it is important to note that though the D&C Act is a Union legislation, there are certain constitutional limitations in the central drug regulatory framework. With respect to the purview of the federal and the state legislatures, the Indian Constitution lays down three lists—the Union List, where the Parliament can legislate upon, the State List, where the state legislatures exercise the sole authority, and finally, the Concurrent List, where both the Parliament and the State Legislature exercise authority.^28^ The matter of ‘public health’ (and hence drug regulation) comes under the purview of the state legislature.^29^ As a result, the state governments exercise considerable autonomy in setting the rules and enforcing standards.

While we have the Drugs Controller within the CDSCO as the central regulatory body under the D&C Act and the Drugs Rules, 1945,^30^ many of the core regulatory functions are exercised by the state-level SDRAs. In practice, while CDSCO regulates aspects regarding clinical trials and new drug approvals, functions related to regulation of manufacturing and distribution of drugs are carried out by SDRAs.^31^ And since the CDSCO and the SDRAs work independently of each other, the former does not exercise any vertical control over the latter. This federal angle of the Indian public health system is often ignored in scholarship on the stem cell regulatory framework.^32^ This has often led to broad calls for new laws, centralized registry, and more stringent measures, without acknowledging the power-sharing dynamics between the federal and regional regulators.

**Figure 1:**
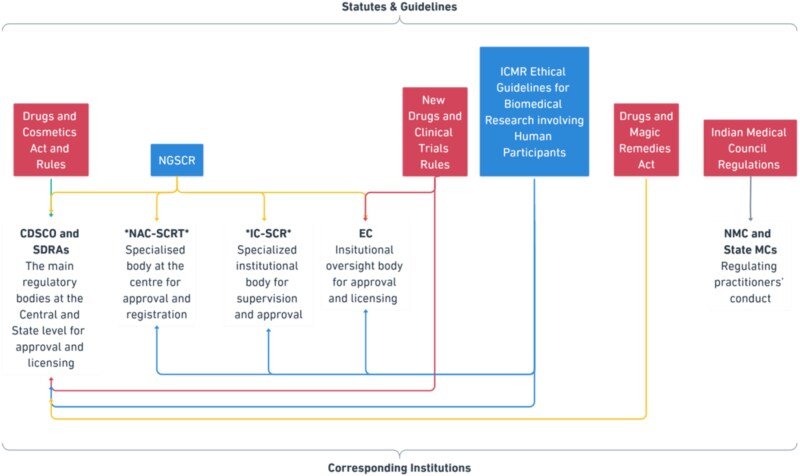
The Regulatory Framework. The red box depicts statutes (enforceable), and the blue box depicts guidelines (unenforceable). CDSCO, Central Drugs Standards Control Organization; IC-SCR, Internal Committee for Stem Cell Research; EC, Ethics Committee; ICMR, Indian Council of Medical Research; MCI, Medical Council of India; NAC-SCRT, National Apex Committee for Stem Cell Research and Therapy; NGSCR, National Guidelines for Stem Cell Research. ^*^NAC-SCRT and IC-SCR are no longer active institutions.

Before proceeding to the next section, it should be noted that the ‘Indian regulatory framework’ refers to the set of legislations and guidelines, and the corresponding institutions which govern stem cell research and allied activities in India. A high-level overview of the framework is provided in Fig. 1.

The role of these institutions and statutory instruments or guidelines is distributed across various areas in stem cell research, namely, clinical trials, therapy, use of hESCs, stem cell banking, and advertisements of therapies/products. Unfortunately, the information available in the public domain is scarce and insufficient to draw a complete picture on the approval and licensing procedures.^33^ However, through additional information obtained via Right to Information (‘RTI’) queries, the subsequent sections aim to provide an overview of the framework.^34^

### II.A. Clinical Trials

According to the World Health Organization (WHO), ‘clinical trials are a type of research that studies new tests and treatments and evaluates their effects on human health outcomes’.^35^ Stem cell–based clinical trials are necessary for the expansion of therapeutic application of stem cells, and this is an area of research that promises cure for serious diseases.^36^ With 117 clinical trials involving stem cell–based intervention registered in India,^37^ regulation of clinical trials occupies an important position within stem cell governance.

**Figure 2:**
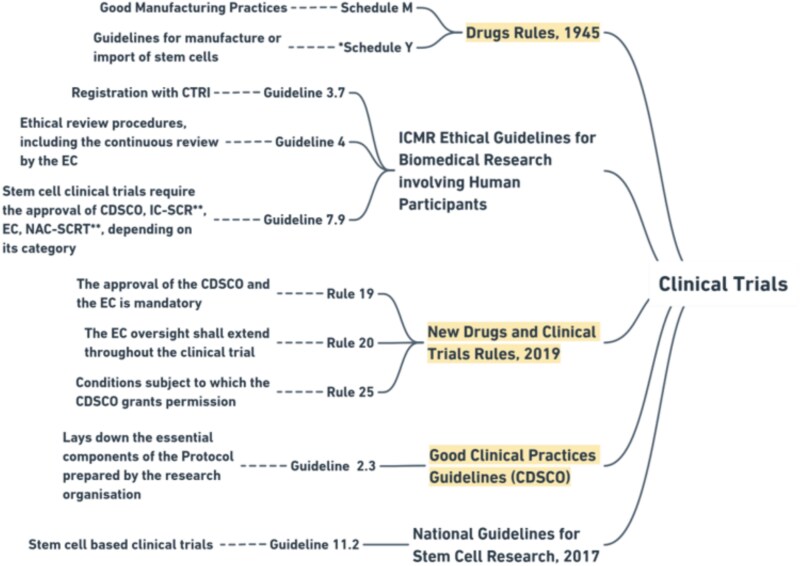
The statutory framework governing clinical trials in India (the highlighted elements are the ones which are enforceable by respective agencies through direct or indirect means). This figure represents only a few important provisions among the relevant ones. ^*^The New Drugs and Clinical Trials Rules, 2019 overrides the Schedule Y guidelines. ^**^IC-SCR and NAC-SCRT are no longer active institutions.

As evident from Figs 1 and 2, organizations conducting clinical research are required to adhere to a catena of laws and guidelines. Clinical trials are regulated by the central-level laws—most importantly, the Drugs Rules, 1945 (‘Drugs Rules’) and the New Drugs and Clinical Trial Rules, 2019 (‘CT Rules’). These rules mandate a multi-tier approval process for different types of clinical trials.^38^ According to the CT Rules, the approval of an institutional body called ‘Ethics Committee’ (EC) is the first step before conducting clinical trials.^39^ The institution conducting the trial has to constitute an EC and register it with the CDSCO.^40^ The EC reviews the proposals for clinical trials, periodically supervises the conduct of clinical trials after the approval, oversees compliance with the Good Clinical Practices (GCP) standards prescribed by the CDSCO, and reports to the CDSCO in case of adverse consequences.^41^ From a simple perspective, ECs act as institution-level nodes of CDSCO.

**Figure 3:**
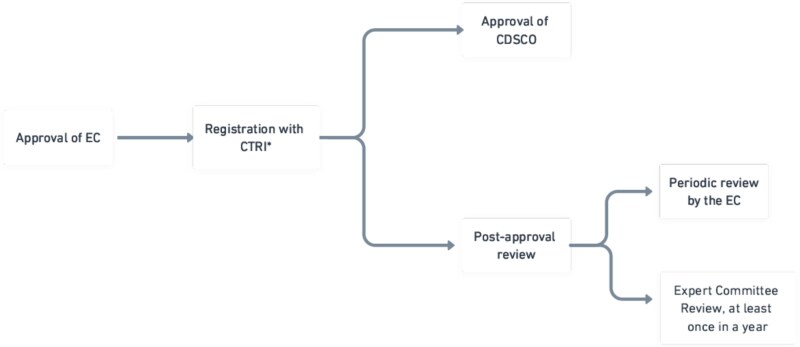
A brief representation of the clinical trial approval process. ^*^CTRI, Clinical Trials Registry—India.

Once the EC approves it, the organization has to make an application for grant of permission to the CDSCO through Form CT-04 under the CT Rules^42^ and/or^43^ Form 44 under the Drugs Rules.^44^ The CDSCO grants permission under Rule 22 of the CT Rules based on various factors mentioned in Rule 122DAC of the Drugs Rules, including the adherence to the GCP, and compliance with the approved protocol.

As evident now, the approval process features a certain degree of regulatory audit. In the first level, the EC is required to monitor the clinical trial site. In addition to this, the CDSCO can authorize officials to conduct on-site inspections even without prior notice.^45^ Also, according to a notice of the CDSCO in 2013, the zonal offices of the CDSCO are required to constitute an expert committee for the conduct of inspections of sites under their jurisdiction at least once a year.^46^ This ensures that the organization adheres to the required rules and regulations even after the permission is granted.

From Rule 122DA of the Drugs Rules, one can also understand that there are three phases to clinical trials, each of which requires separate permission from the EC and the CDSCO.^47^ In certain cases—ie when the clinical trial intended for academic purposes is based on an already approved drug formulation, and it only deals with any new indication or new route of administration or new dose or dosage form of that drug—the approval of the CDSCO is not required once the EC green flags it.^48^

Once approved by the EC and the CDSCO, the next step in line is to register the clinical trial with the national-level registry of clinical trials called the Clinical Trials Registry of India (CTRI), set up in 2007.^49^ Since 2018, it has been mandatory to register a clinical trial before the enrolment of the first patient.^50^ This step ensures that the details of clinical trials are publicly available, facilitating transparency and public awareness and preventing repetitive studies.^51^

Regarding stem cell–based trials, we also have the National Guidelines on Stem Cell Research, 2017 (NGSCR 2017), jointly released by the ICMR and the DBT.^52^ It covers a wide array of issues ranging from stem cell therapy to banking. The first version of the guidelines was released in 2007, titled the ‘Guidelines for Stem Cell Research and Therapy’.^53^ The subsequent versions were released in 2013 and 2017 but without the term ‘therapy’ in their titles.^54^ In addition to the NGSCR, ICMR released the ‘National Ethical Guidelines for Biomedical and Health Research Involving Human Participants’ in 2017, which sets out the measures that the organization or researcher needs to adhere to while conducting clinical trials on human subjects.^55^

While these guidelines provide details concerning the approval procedure, there is no binding mechanism to ensure adherence to these guidelines. On one hand, the DBT and the ICMR, which issued the guidelines, do not have any enforcement powers to mandate compliance.^56^ And, on the other hand, the NGSCR guidelines do not have any legislative backing^57^—that is, neither the Drugs Rules nor the CT Rules particularly require compliance with the NGSCR guidelines for stem cell–based clinical trials.

Specifically, the NGSCR 2017 mandates the approval of an ‘Institutional Committee on Stem Cell Research’ (IC-SCR), registered with the ‘National Apex Committee on Stem Cell Research and Therapy’ (NAC-SCRT).^58^ However, this two-tier arrangement of IC-SCR—NAC-SCRT was, dissolved in March 2024, citing difficulties in implementation.^59^ Before its dissolution, IC-SCR was to NAC-SCRT, like how EC is to CDSCO.^60^ The IC-SCR used to be an EC-like institutional level body associated with the evaluation of clinical trials. It was responsible for reviewing the research protocols, evaluating the compliance with the guidelines, and maintaining a record of the research activities of the institution.^61^ Since the NGSCR has not been updated since 2017, the IC-SCR and the NAC-SCRT still appear in the guidelines.

The NGSCR classifies stem cell research into three categories—permissible, restrictive, and prohibited.^62^ However, clinical trials, irrespective of the category they fall in, require the approval of the EC and the CDSCO.^63^ Previously, it was doubtful whether the approval of IC-SCR is mandatory in practice, as the Drugs Rules and the CT Rules are silent on the same, and the NGSCR Guidelines are not legally enforceable otherwise.^64^ With the dissolution of the IC-SCRs and the NAC-SCRT, this ambiguity no longer exists. Currently, clinical trials require the approval of EC and registration with the CDSCO.^65^

According to the NGSCR 2017, clinical trials using human stem cells are required to adhere to good manufacturing practices (GMPs) in Schedule M of the Drugs Rules,^66^ CDSCO’s GCP, and the ICMR Ethical Guidelines for Biomedical Research involving Human Participants^67^ in addition to other provisions of the CT Rules, the D&C Act, and the Drugs Rules.

While that is about the conduct of clinical trials, there are also stipulations regarding the qualification of the researchers. The medical professional undertaking the clinical research must be registered with the National Medical Council (NMC) and must have an NMC-approved postgraduate qualification specializing in the domain area of the clinical research.^68^

### II.B. Stem Cell Therapy

#### II.B.1. Bone Marrow Transplantation

In India^69^ and in many other countries,^70^ the only recognized stem cell therapy is the bone marrow transplantation (BMT), also known as the hematopoietic stem cell transplantation (HSCT). The application of all other stem cell interventions is considered experimental and is restricted to clinical trials.^71^ The reason being that the safety and the therapeutic efficacy of other forms of stem cell therapy are still doubtful and unclear.^72^

While the BMT comes under the umbrella term of stem cell therapy, the NGSCR is considered inapplicable over it as BMT is now a ‘well-established’ and standard form of treatment.^73^ The ICMR released separate guidelines for BMT, termed the ‘National Guidelines for HSCT’ in 2021.^74^ The guidelines classify the application of BMT into four categories—‘standard of care’, ‘clinical option’, ‘developmental’, and ‘generally not recommended’.^75^

In the case of indications coming under the first category, the BMT is considered a standard option and is carried out without further oversight.^76^

For indications under the category of ‘clinical option’, the application of BMT is considered risky. The patients must be thoroughly briefed about the benefits and risks of the treatment, and the treatment must be carried out only in a specialized setting by well-experienced practitioners.

The third category is for cases where the application of the BMT is considered ‘developmental’ due to ambiguity regarding the role of HSCTs for certain indications. In such cases, the BMT must be preceded by the approval of the transplant protocol by a local ethics committee.

Apart from the above cases, there are also indications for which the BMT is not recommended, and such indications fall within the fourth category. These also include cases where further research is required to ascertain the efficacy of BMT. The guidelines call for more research into the application of BMT for indications under the third and fourth categories.^77^

#### II.B.2. (Unproven) Stem Cell Therapies Still under Research

Currently, clinical trials are being conducted across the globe with the hope of leveraging stem cell therapy for a host of chronic or grave diseases.^78^  Table 1 provides a list of conditions^79^ and comments regarding the potential stem cell treatments.

**Table 1:** Potential stem cell therapies for specific conditions. The comments are based on the analysis presented and recommendations made in the ICMR’s Evidence-Based Status of Stem Cell Therapy for Human Diseases^80^

**S.No.**	**Specific conditions**	**Comments**
1	Orthopedic conditions and injuries	The clinical trial results are encouraging. However, with the available evidence, stem cell therapy is not recommended as the standard treatment.
2	Spinal cord injury (SCI)	While animal experiments and pre-clinical studies have shown positive results, the result of the clinical trial is not sufficient to confirm its effectiveness. Hence, not recommended for standard therapy.
3	Critical limb ischemia (CLI)	The existing studies and experts’ analysis do not support the use of stem cell therapy as the standard treatment. There is, however, a stem cell–based product, stempeucel®, that has been granted a market license by the CDSCO. It can be used only when the traditional treatment is not successful or possible.
4	Duchenne muscular dystrophy (DMD)	Due to technical limitations concerning the use of stem cells for this condition, studies do not support the use of stem cell therapy. One of the technical limitations is that stem cell therapy for DMD cannot be administered without genetically engineering stem cells to correct gene defects—a technique that is still under study.
5	Autism spectrum disorder (ASD)	Existing studies do not support the use of stem cell therapy for ASD, either as a replacement for or as an addition to the current therapies.
6	Lysosomal storage disorder (LSD)	The use of stem cell therapy is not recommended due to the limited number of clinical trials and weak evidence base. However, with proper supervision of experts, the use of HSCT is permitted in Hurler syndrome for children below the age of 2 years.
7	Amyotrophic lateral sclerosis or motor neuron disease (MND)	The existing studies do not support the usage of stem cell therapy as a standard treatment.
8	Stroke	Though there are a few clinical trials studying the use of stem cells for treating (ischemic) stroke, the available evidence is inadequate due to various reasons. Hence, the use of stem cell therapy is not recommended.
9	Multiple sclerosis (MS)	The existing studies do not support the use of stem cell therapy as an addition to or replacement of standard treatments.
10	Heart failure	The clinical trials have not yielded consistent results so far. Hence, stem cell therapy can be administered only within the framework of clinical trials, but not as standard treatment.

**Figure 4:**
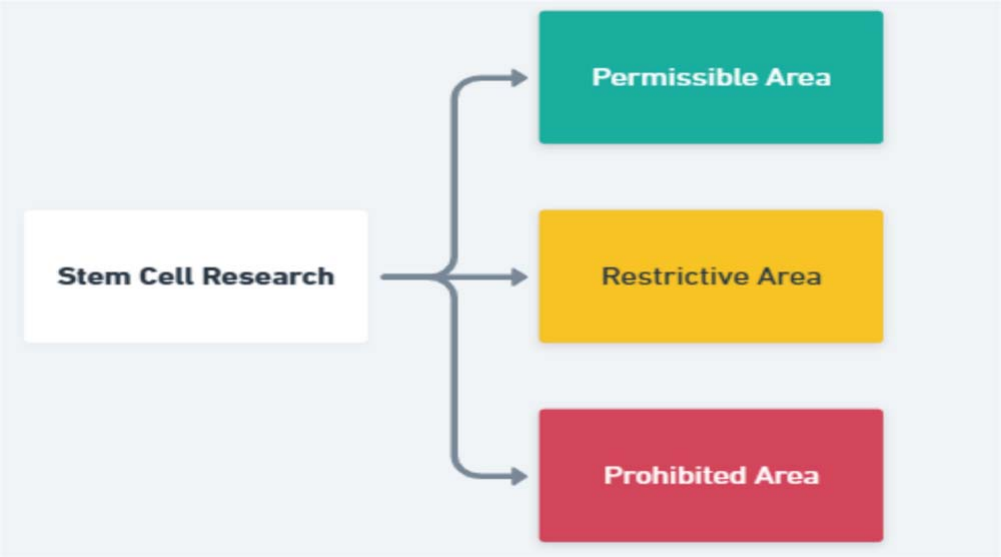
Categories of Stem Cell Research under the NGSCR 2017.

In essence, stem cell therapies other than BMT are still in the experimental stage. There is a need for further research to establish their safety and efficacy. According to the NGSCR 2017, the investigator ought to apply to the ICMR when the clinical trial appears to be promising in terms of a potential therapy.^81^ ICMR will examine the tenability of the claims in consultation with the experts.^82^

Clinicians administering unproven therapies outside the framework of clinical trials can be held liable for ‘misconduct’^83^ and shall face appropriate disciplinary actions under Chapter 8 of the IMC Regulations.^84^ In the event of any injury caused to the patient, they can be held liable under the Bharatiya Nyaya Sanhita, 2023,^85^ which is the principal Indian penal law and the Consumer Protection Act, 2019.^86^ Further, the laws prohibit clinicians and hospitals from advertising such treatments; more on this can be found later in this part.

### II.C. Approach Toward the Usage of Human Embryonic Stem Cells

The use of hESC for stem cell research is a matter of ethical and legal debate in many jurisdictions.^87^ The hESC is a type of pluripotent stem cell, meaning that they have the innate ability to regenerate itself indefinitely into distinct cell types, including specialized cells. In other words, clinical applications involving hESCs can potentially treat several incurable degenerative diseases that were incurable otherwise.^88^

However, the clinical applications involving hESCs are considered controversial due to underlying ethical questions pertaining to the onset of personhood.^89^ The proponents of embryonic stem cell research argue that an embryo is not a person, and it is ethical to use embryos for the purpose of research and clinical applications.^90^ On the other hand, the opponents argue that an embryo is a part of the continuous development of a ‘person’ and that destroying embryos on the basis of potential benefits is unethical and immoral.^91^ However, it appears that the development of ‘induced pluripotent stem cells’ (iPSCs) has addressed some of the above concerns.^92^ iPSC is a type of stem cell that can be obtained from non-embryonic sources but can be reprogrammed to achieve the features of an embryonic stem cell.^93^ As a result, iPSC enables a researcher to avail the benefits of embryonic stem cells without destroying any embryo.

The Indian framework permits both the creation of embryos and the limited usage of surplus embryos for stem cell research.^94^ The Assisted Reproductive Technology (Regulation) Act, 2021 allows research on human gametes and embryos ‘within’ India, and such research has to comply with the guidelines issued by the ICMR.^95^ The NGSCR Guidelines issued by the ICMR and the DBT categorize areas of research as ‘permissible’, ‘restrictive’, and prohibited’ based on the source of the stem cells and a few other factors.^96^ The establishment of new hESC lines from spare embryos falls within the permissive category.^97^ On the other hand, the creation of human embryos *in vitro*, for the purpose of deriving hESC lines, is placed under the ‘restrictive category’, requiring the investigator to prove that the creation of embryos is necessary for conducting the said study.^98^ This approach, which may be qualified as progressive by some, differs from that of other countries, including many European countries.^99^ The Guidelines also conform to the universally accepted ‘rule of 14 days’—that is, human embryos cannot be used for derivation of stem cell lines if 14 days have passed after fertilization, or if a primitive streak has appeared on the embryo, whichever is earlier.^100^ This is categorized as a prohibited area of research.^101^

### II.D. Stem Cell Banking

**Figure 5:**
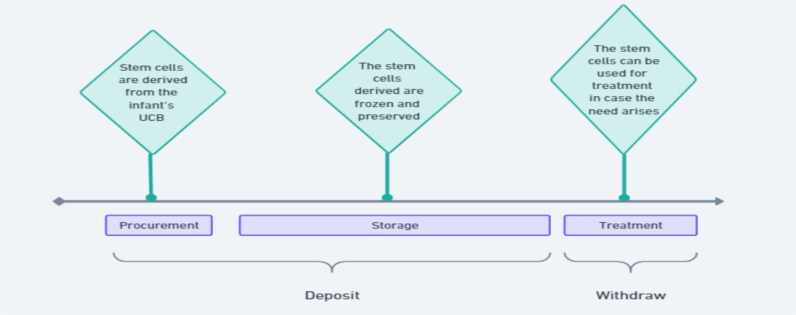
The general model of stem cell banking in India.

Stem cell banking is an emerging business in the stem cell industry; it can be plainly defined as ‘a procedure wherein the cells isolated from various sources are collected, proliferated, stored and preserved for their future use’.^102^ India permits only umbilical cord blood (UCB) banking—which is subjected to the approval and license of the CDSCO.^103^ The ICMR banned banking in all biological materials other than UCB in 2017 (see Figure 5 for the general model of functioning of the UCB banks).^104^

The 2011 amendment to the Drugs Rules brought UCB banking under its ambit.^105^ For setting up a UCB bank, the applicant has to file Form-27F under the Drugs Rules with the CDSCO^106^ and it subsequently grants the license on the basis of on-site inspection.^107^ UCB banking can be private or public. In public banking, people donate UCB for future use by any patient. In private banking, the donor deposits the cord blood in the bank, which can be retrieved in the future at their will for their own use.

Various private enterprises offer the storage of cord blood, promising future therapeutic use. Such advertisements can be misleading. According to the NGSCR, the therapeutic application of stem cells derived from the UCB shall be limited to HSCT-related indications set out in Annexure III. In other cases, the stem cells derived from UCB shall be used only for clinical trials.^108^

A simple web search on ‘stem cell banking’ will expose one to umpteen numbers of private ventures advertising the benefits of UCB banking and positing exaggerated and unverified claims of the potential of stem cells derived to cure ‘life-threatening’ diseases. The private players often market UCB through blanket statements that bely the actual utility of private stem cell banking,^109^ a practice that exploits the lack of sufficient awareness among the lay people.^110^

Based on complaints of such malpractices, the ICMR issued the ‘Guidelines for Umbilical Cord Blood Banking’ in 2023, which lays down the general standards that a bank must follow. It provides for measures to deal with use of misleading advertisements and publicity by the banks.^111^

### II.E. Misleading Advertisements and Claims

It is not uncommon to see clinics and hospitals reaping exorbitant profits by offering stem cell treatment for certain diseases, under the guise of clinical trials.^112^ There have also been instances where misleading advertisements used the photographs or addresses of prominent scientists to justify their claims.^113^ Three principal statutes address the issue of illegal advertisements and the popularization of stem cell therapy,^114^  *viz.*, the Drugs and Magic Remedies (The Objectionable Advertisements) Act,^115^ the Indian Medical Council (Professional Conduct, Etiquette and Ethics) Regulations, 2002 (‘IMC Regulations’)^116^, and the Drugs Rules (specifically note, Schedule J).^117^ These legislations provide for a range of punishments varying from cancellation of license to imprisonment and are enforced by respective regulatory institutions.^118^

The DMR Act and the Drugs Rules empower the Directorate General of Health Services and the CDSCO to take steps in coordination with SDRAs against misleading advertisements.^119^ In the case of UCB banks, a complaint regarding misleading claims can be sent to the nearby zonal branch of the CDSCO.^120^ Additionally, regulation 6.1 of the IMC Regulations precludes clinicians from advertising themselves through any mode.^121^ The NMC and the Medical Councils (MCs) of the respective states have the power to revoke the license of the clinicians in case of violation of the Regulations.^122^ Further, it is possible to take steps under the Consumer Protection Act, 2019^123^ in case of misleading advertisements concerning stem cell products and therapy. Also, according to Rule 106 of the Drugs Rules, advertisements promising cure for or prevention of diseases mentioned in Schedule J of these Rules are prohibited.

The deterrent effect of this multifaceted regulatory mechanism against misleading advertisements and other forms of exploitation of potential consumers of ‘stem cell treatment’ is contingent upon the timely enforcement of these statutes and the proper exercise of the aggrieved party’s right to file a complaint.

## III. A CRITICAL APPRAISAL OF THE INDIAN REGULATORY MECHANISM

The previous section provided a high-level overview of the Indian regulatory framework on stem cell research, clinical trials, and therapy. Given this background, the following section critically analyzes the extant framework.

Firstly, it is important to highlight the crisis of unproven stem cell therapies that we are facing. Since the 2000s, the popularity of untested stem cell interventions has surged over the years, across jurisdictions.^124^ By exploiting the vacuum in the Indian regulatory framework, practitioners and commercial enterprises have taken upon the (unconfirmed) promises of these therapies to lure the affluent and the growing middle class in India, and often times, the medical tourists from developed countries.^125^

Exploitative stem cell tourism has grown as a natural upshot of the proliferation of unscrupulous players. Stem cell tourism, a term that became popular over the past two decades, indicates the practice of patients travelling to avail unproven stem cell therapies based on online, direct-to-consumer advertisements.^126^ It involves desperate patients afflicted with serious conditions, who often seek affordable treatment in foreign countries, trying to exhaust all means available to them.^127^ The glimmer of unproven stem cell therapies touted as the all-round panacea by clinics has attracted a fairly large share of tourists to low- and middle-income countries like India, Thailand, Dominican Republic, and Mexico due to ease of travel and lower costs.^128^ Nevertheless, illegal stem clinics are also propping up in developed countries, including the USA, Japan, and Australia, by way of aggressive marketing.^129^

In India, the lax implementation, the consequent proliferation of illegal clinics, and the concerning trajectory of stem cell tourism lie in inadvertence and complacence than intentional relaxation of laws for attracting foreign investments.^130^ In other words, the growth of stem cell tourism in India lies in the face of loose regulations and lack of effective implementation of existing laws.^131^

Unscrupulous players in the industry have taken advantage of the overlapping, intricate, and porous regulations to further their business,^132^ a phenomenon that can be termed ‘regulatory arbitrage’. In a broad sense, regulatory arbitrage refers to the act of entities designing their transactions or activities in such a way as to take advantage of the gaps in the regulatory framework.^133^ For example, it is possible for a clinic to not comply with certain aspects of the ICMR guidelines on stem cell research and human-based clinical trials.^134^ They can argue that they are not part of any legal mandate set by the statutes.^135^ This has given profit-oriented clinics and practitioners the confidence to actively commercialize stem cell therapy and lure patients from India and abroad.^136^

This is not to suggest that the sky is all dark and cloudy here. In the early 2010s, we faced the fundamental problem of what Tiwari et al. describes as jurisdictional ambiguity—that is, the confusion over who possesses the authority to regulate stem cells and whether it is within the remit of the DCGI/CDSCO.^137^ However, with the amendments in the statutes over the subsequent decade, this ambiguity regarding DCGI’s/CDSCO’s jurisdiction over the issue has been resolved.^138^ Hence, it would be wrong to classify the legislature and the regulator as completely non-responsive. But, still, there are various factors on which the system has to improve.

Having set the context, I take up some of the critical issues that have led to the growth of unproven therapies in the upcoming paragraphs. It is not possible to regulate stem cell tourism without addressing these lacunae.^139^ Steps must be taken in this direction as the growth of stem cell tourism in the current manner is not only fatal^140^ to the vulnerable patients^141^ and to the reputation of India’s medical sector but is also at the cost of development of stem cell science.^142^

### III.A. The Lack of Legislative Recognition for Critical Guidelines

The NGSCR issued by the ICMR and DBT does not have any legislative basis. Before 2019, one had to ensure compliance with the Schedule Y Guidelines to conduct a stem cell clinical trial.^143^ Now, instead of Schedule Y, stem cell trials are governed by the guidelines in the CT Rules. For example, Rule 25(vi) of the CT Rules mandates compliance with the GCP Guidelines.^144^

The point I am trying to make here is that, unlike the GCP guidelines, there is no provision in the statutes that mandates compliance with the NGSCR guidelines. No provision in the general law, ie, Drugs Rules and the specific law, ie, the CT Rules, demand obedience to the NGSCR guidelines as a prerequisite for stem cell trials. The implication is that violation of the NGSCR guidelines does not attract legal sanctions.^145^ It is also quite ironic that both the ICMR and the DBT, institutions responsible for promoting and facilitating biotechnology and biomedical research, do not have the power to enforce their guidelines and penalize the violators.^146^

It appears that practitioners have exploited this loophole in the past.^147^ One can see umpteen number of cases where stem cells are used commercially for unproven therapies, though the prescribed rule is that they cannot be used outside an approved clinical trial setting.^148^ This creates an ironic situation where, despite having ostensibly ‘strict guidelines’^149^ for the regulation of stem cell therapy, we are witnessing an unregulated sprouting of clinics promoting and providing stem cell therapies.^150^

The most pertinent example is the decision of the Delhi High Court in 2023 in *Dalip Kumar v Union of India.*^151^ Here, the Court had to permit a hospital to continue with the ongoing (unproven) stem cell therapy as part of the treatment for autism spectrum disorder (ASD) in two children, citing the potential hazards in abruptly ending the treatment. The Court went on to acknowledge that there was no law expressly banning the use of stem cell therapy for ASD.^152^

In the previous discussion on stem cell therapies, we saw that the ICMR clearly suggests against the use of stem cell therapy for ASD.^153^ Firstly, the question is how did the hospital *initiate* the stem cell therapy in express violation of the ICMR guidelines. Secondly, as understood from the Court’s reasoning, we lack a consolidated law that clearly delineates what is permissible and what is not permissible. These guidelines require statutory recognition to make it enforceable and thereby engender better compliance.^154^

### III.B. The Systemic Procedural Ambiguity in the Approval and Oversight of Clinical Trials

Patra and Faulkner have pointed out the ‘increasing complexity of regulations’ as one of the factors that have enabled the players to circumvent scrutiny in India.^155^ But, from a legal prism, what makes these regulations complex? The answer lies in the often-incomplete information on the procedural aspects due to doubts on how the soft law interacts with the hard law.

For example, the D&C Act and Rules and the CT Rules lay down a definite application procedure for conducting clinical trials. Parallelly, the NGSCR Guidelines also lay down a procedure for the same. The former set of rules does not conflict with the latter guidelines, but the latter guidelines posit a few additional stipulations that the organization needs to adhere to. For instance, the NGSCR Guidelines stipulate that the clinical trials should comply with the ICMR Ethical Guidelines.^156^ However, the above compliance requirement is not mentioned in the provisions concerning clinical trials in the Drugs Rules and the CT Rules and can, therefore, cause confusion among the researchers.

Before the dissolution of the NAC-SCRT, there was a similar issue with regard to the requirement of approval from the IC-SCR. While the Drugs Rules and the CT Rules do not mandate approval from IC-SCR, let alone the constitution of a body called IC-SCR, the NGSCR Guidelines treated the same body as an important regulatory actor.^157^ With their dissolution, this issue has been solved—still, a large number of provisions in the NGSCR continue to deal with the oversight of IC-SCRs and the NAC-SCRT.

The above discussion indicates the absence of a clear and consistent regulatory environment, which is a prerequisite for a vibrant stem cell research ecosystem. As a result, an investigator going through the approval process would be faced with the dilemma of which stipulations to comply with. The ICMR Ethical Guidelines and the CT Rules were introduced in light of the under-regulation of clinical trials.^158^ However, despite being well intended, the regulatory framework appears nebulous in its current form.

This procedural ambiguity is harmful to the development of research activities. For example, the ambiguous network of rules, regulations, and guidelines on research approvals has disproportionately affected governmental and reputed research institutions while benefitting certain commercial private players, as highlighted by a member in the House of the People of the Indian Parliament.^159^

Additionally, the issue here is not just about the absence of any specialized provision or legislation for stem cell research. It is also about the long-term relevance of the NGSCR Guidelines if it is not mandatory in the first place. This regulatory uncertainty is one of the primary issues that needs to be addressed.

### III.C. Lax Implementation of the Law Despite Blatant Violations

It appears that even enforceable aspects of the regulatory framework are under-implemented.^160^ Barring the ambiguities in the framework, a neutral and bare reading of the legislations and guidelines does give an impression of a fairly well-designed system in place.^161^ For example, there are multiple laws that penalize misleading and fraudulent advertisements.^162^ We have a set of legislations consisting of the IMC Regulations (2002), The Drugs and Magical Remedies (The Objectionable Advertisements) Act (1954), the Drugs and Cosmetics Act (1940) and Drugs Rules (1945), Bharatiya Nyaya Sanhita (2023), and Consumer Protections Act (2019), which enable a person to seek civil and criminal remedies.^163^

However, in praxis, it is doubtful whether the associated institutions effectively exercised the powers available under these legislations, with the regulatory framework yielding to various socio-political factors.^164^ For example, Nutech MediWorld, led by Dr Geeta Shroff, is a private player that continues to advertise regenerative therapies for ‘incurable conditions’,^165^ despite it having being brought to the center of various controversies and disputes before.^166^ After all, India is currently in a situation where we are doubtful whether even the public institutions, let alone the private players, are complying with the framework in place.^167^

### III.D. The Inefficiency of the CDSCO due to Burdensome Separation of Duties

Another major drawback is the fragmented style of regulation in India. From the previous discussion, it is apparent that the framework involves multiple players functioning in overlapping areas, with no set workflow. This has not only led to uncertainty and confusion among clinicians but also created doubts regarding distribution of functions among the regulators themselves.^168^

Notably, parallel to vagueness in distribution of duties, there also exists ‘jurisdictional overload’. On one hand, the CDSCO is entrusted with a long list of functions. On the other hand, the CDSCO is not supported by sufficient resources.

**Figure 6:**
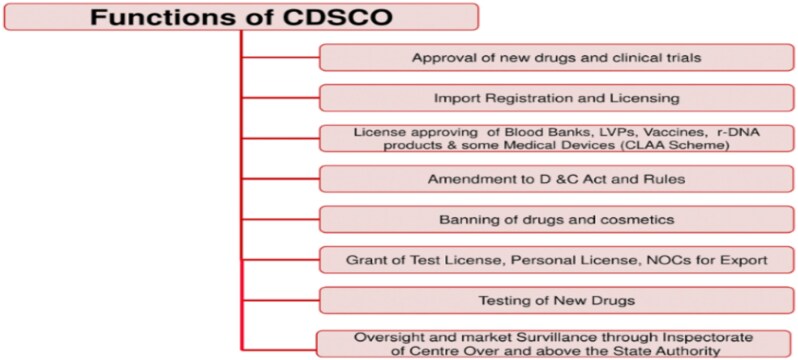
Functions of the CDSCO as provided on their website.

As already noted, CDSCO is the primary actor whose jurisdiction extends over a wide aperture within the arena of stem cell research. However, CDSCO is a general regulatory body whose purview is not limited to stem cell research but extends to the whole catena of drugs and clinical trials in India.^169^ The broad functions of CDSCO, as provided on its website, are elucidated in Fig. 6. An institution like the CDSCO, which is entrusted with a wide range of matters within the field of drug regulation, is charged with the duty of regulating a highly niche field of biomedical research that is growing at an unprecedented rate.

Secondly, these broad and hefty functions of the CDSCO coincide with the fact that it is abysmally understaffed and low in resources.^170^ The 59th Report of the Department-Related Parliamentary Standing Committee on CDSCO highlighted the poor enforcement of the rules due to low capacity, including the disproportionately low number of inspectors to monitor the system.^171^

Further, the coordination between the CDSCO and the MoHFW is lacking. The most striking example that evidences this is the 2016 ban imposed by the Central Government on fixed dose combinations—an instance when the Government imposed a ban disregarding the prior approvals of the DCGCI (CDSCO).^172^ All these go on to show that the current institutional arrangement is not equipped well to handle the rapidly changing stem cell market.

## IV. What Can be Done to Improve the Situation?

The previous part dealt with the inherent shortfalls and fault lines in the Indian regulatory mechanism. The subsequent discussion posits tangible reforms which can plausibly address the maladies of the Indian framework.

### IV.A. Consolidation of Laws for Clarity and Uniformity

#### IV.A.1. Why Do we Need a Special Consolidating Law?

The first step to clear and effective implementation of the law is to ensure that the law is accessible, known, and clear. In the case of stem cell regulation, we already saw that the rules are spread out across legislations with little harmony; this makes enforcement and inducing compliance incredibly difficult. The legal problem here is clearly evident from the dilemma faced by the Courts when faced with a dispute over the legality of the treatment—that is, there is no clear law that clarifies which kind of therapeutic application is permitted^173^ and no policy direction on which and what licenses are required for delivering stem cell therapies and what action to take in the case of unlicensed clinics.^174^

To close this gap, the paper advances enactment of a special consolidating legislation to lay down a clear licensing and approval procedure, a distinct categorization of embryonic research, and a clear separation of the roles of the involved institutions. It might indeed appear ironical when I suggest a single law as a solution to the above-discussed complex problem. Here, it is to be noted that such a consolidating law is just a primary step forward toward better regulation of stem cell research. As pointed out by Dr Amit Prasad, the generic argument for a new law to solve the issue of unscrupulous stem cell therapy ignores the nuances of the patients’ needs and the larger socio-political context.^175^ There is a need for a more studied and careful approach to regulating stem cell therapy, keeping in mind the dynamics of patient welfare and support, which is emphasized toward the latter half of this part on reforms.

However, to start with, what we require for better regulation is clarity in the laws—which is necessary not only for the regulators to enforce it but also for the players to comply with it. In the Indian context, we are lacking greatly on that count. Our existing laws also do not conceive a clear demarcation of powers between the state-level SDRAs and the federal-level CDSCO, leading to ambiguity in the regulatory approach. There is also no standard procedure for taking care of the aggrieved patients who might not just be seeking a remedy against the malpractice but also looking for alternative healthcare support. Hence, there is a need for specific legal mechanisms and safeguards^176^ for addressing this issue, which is not feasible to introduce as amendments to the existing drug law.

Enacting a law on this matter is significant for another reason too. Regulation of stem cell research involves morally and ethically contested issues of creation and use of embryos. Therefore, the legal and ethical sides to the issue need to be debated in the Parliament by the representatives of the people to synthesize and develop a policy vision for undergirding the regulatory framework.^177^

Finally, one might be skeptical toward a suggestion for such a specific law when there is no counterpart for it in other advanced jurisdictions like the USA, the UK, or Japan. In the case of the USA, like in India, the regulation of stem cell therapy is handled by the drug regulator, the Food and Drug Administration. The network of laws in the USA creates a gradation of different levels of regulation depending on the level of manipulation of cells and other factors.^178^ The regulators in the USA have also been quite prompt in responding to the unscrupulous players and proactively communicating with the public,^179^ thanks to their strength and capacity in comparison to their Indian counterpart.^180^

In the case of the UK, they have the Human Tissue Act, 2004 and the Human Embryology and Fertilisation Act, 2008, which cover non-embryonic and embryonic stem cell research, respectively.^181^ In the case of Japan, they have the Act on the Safety of Regenerative Medicine, 2014, which deals with stem cell research on a comprehensive scale, starting from research to care and therapeutic application.^182^

The catch here is that the specific legal context in these jurisdictions does not demand a special law for stem cell research but rather incremental amendments to these to adapt to the developments and improvise governance.^183^ Similarly, the need for a special law has to be situated within the bio-legal context of India,^184^ which has been explained in length in the previous paragraphs and sections. What we require is a law that the courts and the regulator can undoubtedly rely on when faced with a complex situation involving various nuances.

#### IV.A.2. What Does Such a Law Entail?

To analyze the characteristics of such a Stem Cell Regulation Act or SCR Act, it is convenient to divide the process into two stages—the stage of law-making and the stage of policy implementation. The stage of law-making involves designing policies and schemes and giving effect to the same through legislation or legislative amendments.

Since ‘public health’ is the legislative forte of the state, constitutional constraints are applicable in enacting a full-fledged SCR Act.^185^ However, from the existing regulatory practice, regulation of clinical trials and new drug approvals still comes under the central purview.^186^ Hence, a national-level SCR Act may still be enacted to govern stem cell–based clinical trials.

So, the SCR Act shall:

- Clearly define stem cells and their derivatives.
- Adopt a risk-based classification of SCPDs, say based on the level of manipulation.^187^
- Categorize embryonic stem cell research as given in the NGSCR 2017.
- Lay down a specific procedure for application for clinical trials, including a flexible yet definite list of standards or guidelines that need to be adhered to. Ideally, the NGSCR 2017, and the ICMR Guidelines on Biomedical Research involving Human Subjects shall be given statutory recognition through these provisions.
- Set up a protective net for the safety and welfare of human subjects in these trials through a rights-based approach through patient disclosure and consent protocols.^188^ If the patient is opting for an unproven therapy in a clinical trial setting, then higher standard of voluntary free informed consent and associated protocols must be set.^189^ Note that patients undergoing experiential therapies in a clinical trial setting should not be charged any amount for ‘treatment’ but is rather voluntary.^190^ In case of injury or death, there should be a provision for interim compensation on an immediate basis.^191^
- Provide a reasonable timeline for the completion of the licensing and approval procedures.
- Provide for periodic inspections of the clinical trial site.^192^ Inspections are highly important, and their significance can be understood if one looks into the USA. Despite having a proactive regulator, one of the factors that has enabled the unscrupulous players to slide through the regulations has been the rarity of FDA inspections.^193^ Also, establish a ‘stem cell research fund’ to enable a harmonized and effective system for supporting research.^194^
- In the case of proven or approved stem cell therapies, specify the minimum qualifications of the clinicians administering stem cell therapy such that they have reasonable experience and training in clinical stem cell science.^195^
- Specify the procedure for approval and licensing of stem cell banks, along with the list of standards and guidelines that need to be complied with.
- Lay down the penalties for violation of the law, with imprisonment in cases where the health of the patient/trial subject is endangered. For example, in the UK Clinical Trials Regulation, it is a criminal offense to conduct clinical trials in violation of the regulations or unscrupulously sell experimental medicinal products without authorization.^196^

Before finalizing the procedure, the penalties and other details in the SCR Act, the government should exhaustively study the current stem cell research environment in India. Questions and matters on stem cell research in India have been raised by the Members of the Parliament an umpteen number of times before, and it has become a recurrent matter of debate in the Parliament.^197^ Yet, it is surprising that no Parliamentary study has been conducted so far to evaluate the concerns and identify the issues plaguing the weak regulatory mechanism—this could be a reason why there is no sufficient data regarding the proliferation of unproven therapies and why this issue is still behind the shadows, away from the ken of the mass. Before framing the law, a comprehensive expert study is critical to gather sufficient data and understand the dynamics of the stem cell research system—it is often the poor understanding of the system being regulated that leads to failure of the laws.^198^

In the field of biotechnology, there is increasing support for complementing hard laws with soft laws to ensure greater flexibility in dealing with the rapid developments.^199^ As already suggested above, the Act can lay down a general framework while mandating compliance with the specific guidelines of the ICMR, which can be amended and updated easily from time to time. The ICMR can also notify best practices that need not be enforced through hard law. Rather, it can incentivize compliance with them by ensuring benefits in the form of funding or other resources.^200^

While law-making is an important stage to be carried out with conviction, the subsequent stage of policy implementation is a harder task demanding astute judgment and continuous evaluation. In the case of lack of effective enforcement, NGOs and public-policy think tanks play a crucial role in pressurizing the Government. Further, the judiciary plays an important role here. The courts have used tools like ‘continuing mandamus’ to direct the agencies to effectively discharge their public duties and cater to the socio-economic rights of the people.^201^ The courts may also call for ‘performance audits’ to review whether the laws and regulations are effective and ably implemented.^202^ Accordingly, the legislature is expected to introduce amendments that address the lacunae in the regulatory framework.

### IV.B. Setting Things Right with Regards to the Institutions

Irrespective of a clear and definite law, things can go wrong if the implementing institutions are ineffective.^203^ As already discussed, the CDSCO is currently an overburdened institution with an ambiguous radius of duties and functions. According to the 59th report of the Department Related Committee on Health and Family Welfare, the CDSCO is extremely understaffed and low in resources.^204^ A comprehensive study funded by the Thakur Foundation in 2019 identified grave issues with the functioning of the CDSCO and the state-level drug regulators in India, revealing a crumbling system with overworked staffs, dysfunctional testing laboratories, and poor record-keeping.^205^ In such a condition, monitoring the enforcement of standards and regularly inspecting the clinical trial sites would remain a distant reality.

Another issue that was discussed above was the lack of proper coordination between the Institutions. We saw the friction in the functioning between the technical role of CDSCO and the receptiveness of the MoHFW. Further, though we have a special sub-division on stem cell–based products within the Biologics division of CDSCO, it is unclear how it participates in the framing of the rules and guidelines. There is a need to establish a stem cell–specific regulatory division with its own workforce within the CDSCO to monitor stem cell–based clinical trials and conduct periodic inspections on the stem cell based clinical trial sites, with a special emphasis on those involving hESCs due to ethical concerns.

The coordination between the CDSCO and the SDRAs is also a grave concern. The SDRAs command regulatory purview when it comes to monitoring the manufacturing process and sale of SCPDS. There is a need to ensure that the practices regarding regulation of manufacture and sale is uniform across the states. In fact, the D&C Act had already envisioned the Drugs and Consultative Committee (DCC) to engender uniformity in the administration of drugs throughout India.^206^ However, the DCC to date still faces imminent challenges in ensuring uniformity.^207^ To ensure uniformity, there is a need for better coordination between CDSCO and SDRAs and between SDRAs themselves. This requires clear and unhindered channel of communication, something which is lacking currently.^208^

In short, there is an imminent need to restructure the CDSCO to ensure better coordination between the constituent bodies. We need to ensure that technical-policy framing or advisory committees like DTAB are staffed with members of apposite expertise and experience that convenes regularly and is updated with the dynamic research atmosphere. There is also a need to bring the ICMR, the premiere research institute on the matter, into the picture. The regulated players may often try to relegate themselves to lower degree of classification by exploiting the outdated classification in the law.^209^ To avoid this, it is necessary for CDSCO to collaborate with the ICMR on the latest developments in science and update the regulations promptly.

Further, a special regulatory division on stem cells should ensure that the rules regarding clinical trials are followed diligently. Since the function of such a division is to ensure ground-level enforcement of the rules, it should have a localized structure, with such divisions being instituted in the state-level drug regulatory authorities as well. For this localized structure to work, the coordination between the CDSCO and the SDRAs is crucial. While it is not constitutionally permissible to create a vertical hierarchy between the CDSCO and the SDRAs, smoother coordination can be facilitated through regular consultation and policy-framing sessions. In fact, not imposing a top–down control might be more favorable since the presence and prevalence of stem cell technology widely varies from one state to another.

Similarly, inspections mainly come under the purview of the SDRAs and the SCR Act being a central legislation would not able to compel the SDRAs to conduct inspections. However, it can enable the CDSCO to lay down non-binding guidelines and standards for inspections. Through consultative meetings between CDSCO and SDRAs, the state inspectors can be trained to follow the uniform guidelines and standards for inspection.^210^

Another issue at play is the regulation of the medical profession. Tiwari et al. has pointed out that enforcement of professional guidelines governing the medical practitioners is an important facet of stem cell regulation.^211^ What is important is that the State MCs should have the capacity to monitor and take disciplinary actions in instances where the practitioner is found to violate the regulations.^212^ From the patients’ perspective, it is believed that an interim compensation mechanism, suggested above, would provide an immediate reprieve in cases of untoward consequences. Again, for this, the dispute resolution infrastructure involving the consumer fora and civil and criminal courts need to be strengthened.

Talks and discussions on restructuring the institutional framework has been active for some time in light of several issues on drug regulation coming into the light.^213^ With the growing burden of protecting the people from unproven stem cell therapies and saving the researchers from inconsistent procedures, the call for reforms is the loudest now.

### IV.C. Establishing a Dedicated Funding Mechanism

On the face of it, the question of better funding might appear out of place in a discussion on regulation. However, the issues of who is responsible for funding and how and on what basis research is funded are all questions that are central to ensuring ‘clarity’ in the institutional framework. A clear and well-defined program is closely connected with the previously discussed need for a set workflow for institutions with clear separation of functions. In addition to the regulatory benefits, a formal funding plan with a defined vision is helpful in incentivizing basic research as well as public welfare targeted research, thereby allowing private entities to take up advanced research.^214^

In India, the government funds stem cell research through institutions like the ICMR, the DBT, and the Department of Science and Technology (DST).^215^ However, there does not appear to be a defined and consistent funding strategy for stem cell research. The funding has remained noticeably inconsistent over the years—in 2019, the total funds released by ICMR under stem cell research for fellowships and ad hoc projects were Rs 4,36,19,531/−; this amount was reduced by 84% in the following year.^216^ However, in 2021, the total funds released for the same purpose were Rs. 3,08,34,008/−.^217^ Parallel to this, the DBT released Rs. 7345.58 Lakh for projects related to stem cells and other areas of regenerative medicine.^218^ There appears to be no reason why the ICMR and the DBT need to fund stem cell research separately; such an unexplainable divide only fuels the ‘bureaucratic competition’, which was already pointed out by another scholar.^219^

Reportedly, in 2005, the DBT’s stem cell task force asked the government to set up special funds for facilitating research.^220^ This proposal for ‘priority fund’ was welcomed by several stakeholders, despite the fact that it did not materialize.^221^ There must be a well-defined model for funding, and there are several global best practices worth closer scrutiny.

The European Union’s (EU’s) ‘Horizon Europe’ is a pertinent model worth discussing. It is a funding program exclusively for scientific research for the period 2021–2027 & the ambit of the program includes stem cell research.^222^ There are also clear rules as to the funding of different types and categories of stem cell research. For example, funding is not available for research that involves creation of embryos for the sole purpose of stem cell procurement.^223^ Whether a research project is to be funded or not is decided based on multiple levels of ethical review.^224^

The establishment of such a mechanism with due emphasis on stem cell research can institutionalize the funding. Further, restricting funding based on compliance with the set standards can incentivize adherence to the soft law and the changing research norms that otherwise might be difficult to enforce through rigid hard laws. A just funding mechanism will also contribute toward democratizing stem cell–based clinical trials that are highly resource intensive and dominated by elite players.^225^ Ultimately, the question is not about investing more. Economists argue that every ‘Rupee’ of state spending costs the Indian taxpayers roughly three times.^226^ The utility of a funding strategy, hence, lies in reducing wastage and repetition while ensuring certainty.

### IV.D. Empathetically Engaging with the Public

As hinted in the previous section on the special law, enacting a new legislation is just the first step in improving the situation. To address the deeper nuances of the issue, one has to see it from the patients’ and clinicians’ perspective as well. While the regulatory vacuum engendered the growth of clinics providing unproven therapies,^227^ the prevalence of stem cell therapies and the people’s trust in them have been entrenched by various social, political, and technical forces. As pointed out by Dr Rohini Kandhari in her ethnographic study, stem cell therapy has become part of the standard treatment in various ways through the process of ‘normalization’.^228^ Stem cell therapy being touted as the inevitable resort (for certain diseases), its promotion by even the established and trusted players, and the apparently ‘simple’ and ‘harmless’ procedure (in comparison with the proven surgical alternatives) have shaped the consumer choice and the integration of stem cell therapy into mainstream medical treatments.^229^

It can be safely posited that the appeal of unproven stem cell therapy with the consumers is mainly based on the information obtained from various personal sources,^230^ including that of social media.^231^ There are also studies that indicate that a large share of the population is unaware about the potential risks and side-effects of unproven therapies.^232^ The incomplete, misleading, and exaggerated information on which people base their decision is the elephant in the room that needs to be addressed. This also includes opinions of practitioners who might be informally approached and might have to advise such patients without much context or expertise.^233^

With the aim of bringing clarity to the stakeholders, the ICMR has released (jointly with the DBT) the NGSCR Guidelines and published the report titled ‘Evidence Based Status of Stem Cell Therapy for Human Diseases’.^234^ These documents, however, are out of a layperson’s reach. The NGSCR is a technical document and is meant for medical professionals engaged in stem cell research. On the other hand, though the report on the status of stem cell therapy was released keeping in mind the public and the patients, it has not received the due attention within the public.

India needs public education campaigns to address the ‘false hope’ on unproven therapies that patients nurture and clinics exploit. The objective of this sensitization drive shall be to remedy the distorted picture of stem cell therapies; it shall aim to convey to the public that the stem cell therapy suggested to them by professionals or close relatives can be just experimental, and not a standard treatment. Ultimately, the public must also be convinced to accept this reality over the rosy picture presented to them by some well-respected and established practitioners.^235^

Further, there must be an effective mode of communication and outreach—the above suggested outreach program can be implemented through advertisements and campaigns using social media platforms.^236^ The influence and authenticity of the well-known experts in the field can also be leveraged to convince the public. The information must be presented in an accessible form so that it is digestible to all sections of the society, including the marginalized groups. This is particularly important as there have been instances where people experiencing poverty have helplessly subjected themselves to clinical trials under the guise of free treatments or for monetary returns.^237^ As a preliminary measure, we can consider adopting the practice in Japan—where the Ministry of Health, Labour and Welfare publicly lists the clinics violating the law, thus not only providing prospective patients and other stakeholders with the information but also deterring the clinics from non-compliance.^238^ Ultimately, sensitization drives ought to go in tandem with the effective enforcement of the law that prohibits the promotion and advertisement of unproven therapies.

While the above steps may be beneficial in educating the patients and clearing the misconceptions and misperceptions around stem cell therapies, it only addresses one side of the issue and the limitations of such an ‘information-centric’ approach have already been pointed out by the literature.^239^ To realize the other side of the issue, one needs to understand the psychology of the patients. As posited by Dr Insoo Hyun, patients who exhaust the standard options may opt for (unproven) stem cell therapies just out of ‘therapeutic hope’—though it may not be rationally justifiable, they may still tend to delve on this option as a possible yet remote solution.^240^

In other words, public awareness campaigns alone may not be enough to prevent the unscrupulous clinics from exploiting the vulnerable patients. In fact, as Dr Hyun suggests, such public campaigns without any steps to improve the condition of such ailing patients may negatively affect their mentality.^241^ This is where the physicians need to pitch in. They need to informally and empathetically engage with the patients and provide the necessary emotional support to ameliorate their distress.^242^ This engagement can be facilitated by patient support organizations that have played an appreciable role in representing and supporting them. Carloyn Heitmeyer has previously cited the example of the Spinal Cord Consumers Group (SCCG) in India as a patient-activist group.^243^ The SCCG, as a community of ‘consumers’, helps the patients by lending emotional support and by educating the ‘consumers’ of the options available to them through collaboration with practitioners. Such a model not only protects patients, or rather consumers, from exploitative players, but also facilitates clinical trials by supporting patients who are willing to take part in experimental studies. The aim of such programs is to empathetically engage with the patients and not to merely classify them as vulnerable people. Further, civil society groups and patient support groups play an important role in supporting the patients, monetarily or otherwise, in pursuing their complaint before the consumer dispute for and the state medical councils.^244^

While Heitmeyer views this type of collaboration as an alternative to broader regulation by the state,^245^ I suggest that this can be placed well within the larger framework of formal regulations. That is, while state-led regulations can focus on the general function of combatting unscrupulous players, patient-led activist organizations can focus on the specific function of providing support and information to the patients and bringing them in level with the industry players. Hence, a robust patient-activist system should complement the public outreach measures to address the issue of exploitation of patients.^246^

## V. CONCLUSION

India is a promising player in the global arena of stem cell research. If it is to contribute systematically into the field, then it is imminent that the regulatory framework is reformed. After analyzing the regulatory mechanism from the perspective of research, clinical trials, and therapy, it is evident that there is a need to rejig the current system for better regulatory clarity and effectiveness. The issues range from the inherent irony between restrictive guidelines and lax implementation, to the jurisdictional ambiguity and institutional overload. The ramifications of these issues are already before us in the form of misleading advertisements on stem cell therapy, proliferation of clinics without license, unethical unprofessionalism hidden under the unenforceability of guidelines, and an inequitable field for budding stem cell researchers.^247^ With the ambiguity in the regulatory framework, which even otherwise is brimming with hectic paper works, the current environment of stem cell research appears very intimidating for nascent players.^248^

It is in this context that the article throws light upon a few steps that can be taken to improve the Indian regulatory mechanism. One of the suggested measures is the introduction of a long-term funding strategy. Like the EU’s Horizon Europe, it can be a plan that covers funding of scientific research generally. However, India may also consider targeting more specific areas like regenerative medicine. In either of these cases, the plan must incentivize compliance with the guidelines and best practices, along with denying funds in instances of aberrations.

Enactment of the SCR Act is another suggestion. Clear expression of the rules in a consolidated format that gives mandates compliance to a specific list of standards and guidelines would go a long way in bringing the much-needed clarity and consistency in the legal system. Along with this, there is an imminent need to restructure the institutional framework—starting from a stem cell–specific regulatory division to better coordination between the central and state regulators, a functional set of institutions is critical to the uniform enforcement of laws. Equally important is the need to improve the capacity of our regulators. The CDSCO at the drug-regulatory level and the State MCs at the profession-regulatory level require greater manpower and resources and an institutional vision to properly deliver their functions. Further, the dispute resolution infrastructure, consisting of the civil and criminal courts and most importantly, consumer fora, must be strengthened to ensure that there are speedy adjudication and award of remedies.

The article also re-emphasizes the need for sensitizing the public and engaging with the patients through civil society groups and patient support groups. As mentioned before, the issue of people falling prey to unproven stem cell therapies is a cauldron of several socio-political factors. A robust regulatory framework may still fail when the public is uninformed and misdirected. From a long-term perspective, basic awareness measures might be insufficient. We need empathetic practitioners who can explain the therapeutic possibilities to a desperate patient for whom stem cell therapy might be a glimmer of hope. Hence, this process requires concerted efforts from a large number of players, from practitioners and public figures to State mechanisms. The caption that underlies all these reforms is that these are to be seen as integrated measures and not as isolated steps. The impact of these reforms is interconnected with each other. Hence, strategic implementation of the reforms is crucial to achieve the desired results.

Ultimately, this inquiry is timely. The ICMR-DBT Guidelines that are followed today are around eight years old. In the realm of science, specifically stem cell science, it is safe to adjudge it as outdated. Hence, the policy-makers must evaluate the existing scenario and ensure that imminent reforms, as suggested in this article, are taken into consideration.

## Footnotes

1 Pew Research Centre, *Stem Cell Research Around the World* (17 July 2008), available at <https://www.pewresearch.org/religion/2008/07/17/stem-cell-research-around-the-world/> (accessed Mar. 26, 2025).

2 Takeshi Soma et al., *Induced Pluripotent Stem-Cell-Derived Corneal Epithelium for Transplant Surgery: A Single-Arm, Open-Label, First-In-Human Interventional Study in Japan*, 404 The Lancet 1929 (2024); Smriti Mallapaty, *Stem Cells Reverse Woman’s Diabetes—A World First*, Nature (26 Sept. 2024), <https://www.nature.com/articles/d41586-024-03129-3> (accessed Oct. 23, 2025).

3 Jonathan M.W. Slack, *What Is a Stem Cell?*, 7 Wiley Interdisciplinary Reviews: Developmental Biology 1, 1 (2018).

4 Min Yang, *Synthetic Human Embryos Let Researchers Study Early Development While Studying Ethical and Logistical Hurdles*, The Conversation (7 Feb. 2024), <https://theconversation.com/synthetic-human-embryos-let-researchers-study-early-development-while-sidestepping-ethical-and-logistical-hurdles-214186>.

5 F.M. Watt & R.R. Driskell, *The Therapeutic Potential of Stem Cells*, 365 Phil. Trans. R. Soc. B 155, 160–161 (2010); Dinesh Boopalan, Ramanan Pandian, and Gokul Kesavan, *Prospects for Stem Cell-Based Regenerative Therapies in India*, 3 StemJournal 11, 13 (2021).

6 ISSCR, *Guidelines for Stem Cell Research and Clinical Translation*, available at <https://www.isscr.org/guidelines> (accessed Mar. 31, 2025).

7 Sanjana Sutar, Revansiddappa Devarinti & Kurubara Amaresh, *National and International Regulatory Requirements on Stem Cell Research—A Systematic Review*, 17 Indian Journal of Health Sciences and Biomedical Research KLEU 16, 18 (2024).

8 Kavita Srivastava, Lily Srivastava & Tanvi Jain, *A Comprehensive Review of Challenges and Opportunities for Stem Cell Research in India*, 16 Perspectives in Clinical Research 3 (2025).

9 New Drugs and Clinical Trials Rules, 2019, r 2(w) (‘CT Rules’).

10 Mohit Kapoor, *[The Viewpoint] Decoding the Legality of Stem Cell Therapy in India*, Bar and Bench (14 Feb. 2022), <https://www.barandbench.com/law-firms/view-point/the-viewpoint-decoding-the-legality-of-stem-cell-therapy-in-india> (accessed Oct. 23, 2025).

11 Originally, only ‘stem cell–derived product’ was included in the definition of new drugs. Later, it was amended in 2022 based on the recommendation of the Drugs Technical Advisory Board (*See* DTAB, *Minutes of the 86th Meeting of Drugs Technical Advisory Board Held on 13.04.2021 at DGHS, Nirman Bhawan, New Delhi*, 13 Apr. 2021, available at https://cdsco.gov.in/opencms/opencms/system/modules/CDSCO.WEB/-elements/common_-download.jsp?num_id_pk=MTM3NA%3D%3D (accessed Apr. 16, 2025).

12 Based on a search conducted in the India Science, Technology and Innovation Portal with the keyword ‘Stem Cells’ (*See* India Science, Technology and Innovation Portal, *Research*, <https://www.indiascienceandtechnology.gov.in/research?field_area_id=All&keyword=Stem%20Cells%20&parent_area_id=All&page=5> (accessed Dec. 15, 2024).

13 India is one of the countries that actively promotes stem cell research and one of the very few countries that allows the use of human embryonic stem cells for research. [*See* Rajarathna, *Advancements in Stem Cell Research: An Indian Perspective*, 17 Annals of Neuroscience 110, 110 (2010)].

14 IIT Guwahati—Press Release, *Multi Institutional Team Led by IIT Guwahati Produced Pluripotent Stem Cells from Skin Cells* (16 Aug. 2023), available at <https://www.iitg.ac.in/iitg_press_details?p=63/multi-institutional-team-led-by-iit-guwahati-produces-pluripotent-stem-cells-from-skin-cells> (accessed Oct. 23, 2025); *Army Hospital R&R’s First Successful Bone Marrow Transplant Rescues 7-Year-Old from Rare Immunodeficiency*, Financial Express (18 Dec. 2023), <https://www.financialexpress.com/business/defence-army-hospital-rrs-first-successful-bone-marrow-transplant-rescues-7-year-old-from-rare-immunodeficiency-3341102/> (accessed Oct. 23, 2025).

15 Though India’s publication share has improved by 5277% between 1990 and 2014, India’s research output amounts to only 1.22% of the global research output. Hence, despite the rise in quantity, the research quality is sub-optimal. [*See* Arpana Pandey & Pranav N. Desai, *Emerging Structure of Stem Cell Research in India: An Analysis of Publication Output, 1990–2014*, 5 Journal of Scientometric Research 49, 53(2016)].

16 For example, rapid expansion of unproven therapies fueled by commercial interests might not sit well with the patience and meticulous characteristics of scientific integrity (*see* Rohini Kandhari, *Good and Bad Stem Cell Research: How India’s Scientists View Providers of Unproven Stem Cell Treatments*, 3 SSM – Qualitative Research in Health 1, 7 (2023)).

17 Shashank Tiwari and Pranav Desai, *Unproven Stem Cell Therapies in India: Regulatory Challenges and Proposed Paths Forward*, 23 Cell Stem Cell Forum 649, 649 (2018).

18 Shashank S Tiwari and Sujatha Raman, *Governing Stem Cell Therapy in India: Regulatory Vacuum*
*or Jurisdictional Ambiguity?* 33 New Genetics and Society 413 (2014); Tiwari & Desai, *supra* note 17; Jobin Jose, Teena George and Aaaron M. Thomas, *Regulation of Stem Cell-Based Research in India in Comparison with the US, EU and Other Asian Countries: Current Issues and Future Perspectives,* 15 Current Stem Cell Research & Therapy 492 (2020); Sandeep Lohmor et al., *Current Status of Regulatory Perspectives on Stem Cell Therapy in India*, 7 Chemical Biology Letters 176 (2020).

19 Generalized arguments in favor of legal and policy reforms in the context of stem cell regulation have been rightly flagged in Amit Prasad, *Resituating Overseas Stem Cell Therapy*, 12 Regenerative Medicine 743 (2017).

20
*See* Rohini Kandhari, *Stem Cell Treatments in India: An Ethnography of Regular Practice*, 40 Medical Anthropology 348, 358 (2021); Alan Petersen et al., Stem Cell Tourism and the Political Economy of Hope (2017); Carolyn Heitmeyer, *Biogovernance Beyond the State: The Shaping of Stem Cell Therapy by Patient Organizations in India*, 36 Medical Anthropology 202 (2017).

21 K.S. Jayaraman, *Indian Regulations Fail to Monitor Growing Stem-Cell Use in Clinics*, 434 Nature 259, 259, (2005).

22
*Id*.

23 Press Information Bureau, *Stem Cell Treatment* (23 Mar. 2018), available at <https://pib.gov.in/Pressreleaseshare.aspx?PRID=1526110> (accessed Dec. 26, 2024). (‘PIB - SCT’).

24
*See* Kapoor, *supra* note 10.

25 Guidelines for Stem Cell Research and Therapy, 2007 (‘GSCRT 2007’); *See* also Brian Salter, *Governing Stem Cell Science in China and India: Emerging Economies and the Global Politics of Innovation*, 27 New Genetics and Society 145, 152 (2008).

26 PIB – SCT, *supra* note 23.

27 Drugs and Cosmetics Act, 1940, No. 23, Acts of Parliament, 1940 (‘D&C Act’).

28 INDIA CONST., art. 246, schedule 7.

29
*Id*., item number 6.

30 Shree Agnihotri & Sumathi Chandrashekaran (Thakur Foundation), *Drug Regulation in India: The Working and Performance of CDSCO and SDRAs*, 11 (2019), <https://www.thakur-foundation.org/TFF%20State%20Drug%20Regulatory%20Authorities%20Capability%20Assessment%20Report.pdf> (accessed Oct. 23, 2025).

31 Nupur Chowdhary et al. (ICRIER), *Administrative Structure & Functions of Drug Regulatory Authorities in India*, 6 (2015), https://icrier.org/pdf/PolicyBrief_1_health.pdf.

32
*See* Kavita Srivastava, Lily Srivastava & Tanvi Jain, *A Comprehensive Review of Challenges and Opportunities for Stem Cell Research in India*, 16 Perspectives in Clinical Research 3, 7 (2025); Sandeep Lohmor et al., *Current Status of Regulatory Perspectives on Stem Cell Therapy in India*, 7 Chemical Biology Letters 176, 181 (2020).

33 The existing scholarly literature—especially those based on the primary documents and information sought from the stakeholders, has to some extent made up for the lack of information in public domain. The ‘public domain’ includes websites of ICMR, Department of Biotechnology (DBT) and CDSCO, along with the guidelines and legislations. See also Sowmya Viswanathan et al., *Overcoming Challenges to Initiating Stem Cell Therapy, Clinical Trials in Rapidly Developing Countries: India as a Model*, 2 Stem Cells Translational Medicine 607, 607 (2013).

34 This task is considered important for two reasons—it will form the basis for pointing out the gaps in the system, and it will be a valuable addition to the apparently outdated existing descriptive literature on the regulatory practices.

35 WHO – Health Topics, *Clinical Trials*, <https://www.who.int/health-topics/clinical-trials#tab=tab_1> (accessed Oct. 23, 2025).

36 Alan Trounson et al., *Clinical Trials for Stem Cell Therapies*, 4 BMC Medicine 1, 2–6 (2011).

37 An ‘Advanced Search’ in the Clinical Trial Registry of India (CTRI) with the string ‘stem cell’ under the ‘scientific title study’ provides a list of 117 clinical trials.

38 CT Rules, 2019, rule 19.

39
*Id*., rule 6.

40
*Id*., rule 8

41
*Id*., rule 11.

42
*Id*., rule 21.

43 It is unclear as to whether an applicant has to fill in both Form 44 and Form CT-04. While the DC Rules mention Form 44, the CT Rules mention Form CT-04 as the form to be submitted to the CDSCO for the application. I did not get any response on filing this as a RTI question. However, based on principle of *generalis specialibus non derogant*, it can be said that the CT Rules (the special law) supersedes the Drugs Rules (general law) in the matters of clinical trials: hence, the application is believed to be through Form CT-04.

44 Drugs Rules, 1945, rule 122DA(2)(a).

45
*Id*., rules 122DAC(1)(g), 122DAC(1)(h).

46 Office of Drugs Controller General (India), *Monitoring of Clinical Trials – regarding* (F.No.12-01/12-DC (Pt.133) 2013).

47 Drugs Rules, 1945, rules 122DA(2).

48
*Id*., rule 122DA(4).

49 Registration with CTRI was made mandatory in 2009.

50 CTRI, *Frequently Asked Questions*, available at https://ctri.nic.in/Clinicaltrials/faq.php#6a (accessed Apr. 16, 2025).

51 M Vishnu Vardhana Rao et al.*, Clinical Trials Registry—India: An Overview and New Developments*, 50 Indian Journal of Pharmacology 208 (2018).

52 Press Information Bureau, *National Guidelines for Stem Cell Research* (21 Feb. 2014), available at <https://pib.gov.in/newsite/PrintRelease.aspx?relid=104095> (accessed Mar. 26, 2025).

53 GSCRT 2007, *supra* note 25; *Id*.

54
*See* Tiwari & Raman, *supra* note 18, 422.

55 National Ethical Guidelines for Biomedical and Health Research Involving Human Participants, 2017 (‘NEGBHR 2017’); Sandeep Lahiry et al., *The National Guidelines for Stem Cell Research (2017): What Academicians Need to Know?*, 10 Perspectives in Clinical Research 148, 151 (2019).

56
*See* also Question Nos 2, 3, and 4 of Appendix 1.

57 See also Brian Hurwitz, *Clinical Guidelines and the Law: Advice, Guidance or Regulation?*, 1 Journal of Evaluation in Clinical Practice 49, 50 (1995).

58 NGSCR, 2017, guideline 11.2.

59 According to the ICMR, the two-tier mechanism consisting of the NAC-SCRT and the IC-SCR faced difficulties with implementation and it ultimately led to its dissolution. (*See* Ministry of Health and Family Welfare, *Dissolving the National Apex Committee for Stem Cell Research and Therapy* (T.11016/02/2023-HR 2024) available at <https://epms.icmr.org.in/extramuralstaticweb/callforproposal/Dissolution_of_NACSCRT.pdf> (‘Dissolution circular’); *also see* Question No.1 in Appendix).

60
*Compare* NGSCR, 2017, Annexure 1 *and* CT Rules, 2019, rule 8.

61 NGSCR, 2017, Annexure 1.

62
*Id*., guideline 8.

63 Earlier, the trials coming under the restrictive category were subjected to additional oversight. In some cases, the approval of NAC-SCRT was required in addition to that of IC-SCR and the EC. However, with the dissolution of the IC-SCRs and the NAC-SCRT, this research categorization makes little difference on the regulatory side. However, there is a possibility that the degree of oversight by EC may vary depending on the category, with stricter EC scrutiny for restrictive clinical trials (NGSCR, 2017, guidelines 8.1, 8.2 and 8.3).

64 The Order dissolving the NAC-SCRT does not mention anything regarding the dissolution of IC-SCRs. However, the RTI response, clarified that IC-SCRs in existing organizations were also dissolved (*See* Question No 1 in Appendix).

65 Dissolution circular, *supra* note 59.

66 NGSCR, 2017, guideline 4.2.1.4

67 PIB, *Stem Cell Therapy* (11 Mar. 2016), available at <https://pib.gov.in/newsite/PrintRelease.aspx?relid=137747#:~:text=This%20mechanism%20of%20additional%20review,on%2029th%20October%202,012> (accessed Mar. 26, 2025).

68 NGSCR, 2017, guideline 9.13.

69 PIB, *supra* note 67; NGSCR, 2017, Foreword.

70 Harvard Stem Cell Institute – FAQ, *What Stem Cell-Based Therapies Are Now Available?*, available at <https://hsci.harvard.edu/faq/stem-cell-therapies> (accessed Apr. 10, 2025); Timothy Caulfield, *Unproven Stem-Cell Treatments Can Be Dangerous. The Hype Needs to Stop*, The Globe and Mail (July 10, 2019), <https://www.theglobeandmail.com/opinion/article-unproven-stem-cell-treatments-can-be-dangerous-the-hype-needs-to-stop/>; NAC-SCRT, *FAQ Stem Cell Research—Stem Cell Treatment*, available at <https://nacscrt.icmr.org.in/faq.ph> (accessed Apr. 10, 2025).

71 NGSCR, 2017, Foreword.

72 Caulfield, *supra* note 70.

73 NGSCR, 2017, Foreword.

74 National Guidelines for Hematopoietic Stem Cell Transplantation, 2021, Annexure 1 (‘HSCT Guidelines, 2021’).

75
*Id*.

76
*Id*.

77
*Id*.

78 International Society for Stem Cell Research—Clinical Resources, *Disease Fact Sheets*, available at <https://www.isscr.org/clinicians/#disease> (accessed Apr. 1, 2025).

79 The list of conditions and the corresponding comments are based on ICMR, *Evidence Based Status of Stem Cell Therapy for Human Beings 2021*, available at <https://www.icmr.gov.in/icmrobject/uploads/Guidelines/1745229591_evidence_based_status_of_stem_cell_therapy_for_human_diseases_v1.pdf> (accessed Oct. 4, 2025).

80
*Id*.

81 NGSCR, 2017, guideline 12.3.

82
*Id*.

83 Indian Medical Council (Professional Conduct, Etiquette and Ethics) Regulations, 2002, reg 7.22.

84
*Id*., chapter 8.

85 Bharatiya Nyaya Sanhita, 2023, sections 106, 125, No.45, Acts of Parliament, 2023 (‘BNS, 2023’).

86 Consumer Protection Act 2019, No. 35, chapters VI, VII, Acts of Parliament, 2019 (‘CPA 2019’).

87 Michael Mintrom, *Policy Entrepreneurs and Controversial Science: Governing Human Embryonic Stem Cell Research*, Australian Political Studies Association Conference 580, 586 (2012).

88 Tandis Vazin and William Freed, *Human embryonic stem cells: Derivation, culture, and differentiation: A review*, 28 Restor Neurol Neurosci. 589, 589 (2010).

89 Bernard Lo and Lindsay Parham, *Ethical Issues in Stem Cell Research*, 30 Endocrine Review (2009), <https://www.ncbi.nlm.nih.gov/pmc/articles/PMC2726839/> (accessed Apr. 25, 2025).

90 Lisa Bortolotti and John Harris, *Stem Cell Research, Personhood and Sentience*, 10 Ethics, Law and Moral Philosophy of Reproductive Biomedicine 68 (2005).

91 Lillian Nwigwe, *Embryonic Stem Cell Research: An Ethical Dilemma*, 5 Voices in Bioethics 1 (2019).

92 Lo and Parham, *supra* note 90.

93 Lei Ye, Cory Swingen and Jianyi Zhang, *Induced Pluripotent Stem Cells and Their Potential for Basic and Clinical Sciences*, 9 Current Cardiology Reviews 63, 63 (2013).

94 PIB, *National Guidelines for Stem Cell Research and Therapy* (27 Feb. 2015), available at <https://pib.gov.in/newsite/PrintRelease.aspx?relid=116030> (accessed Apr. 12, 2025).

95 Assisted Reproductive Technology (Regulation) Act, 2021, §30(2), No.42, Acts of Parliament, 2021; *read with* Assisted Reproductive Technology (Regulation) Rules, 2022, rule 30.2.

96 NGSCR, 2017, guideline 8.

97
*Id*. guideline 8.1.2.

98
*Id*. guideline 8.2.1.

99 Christian Drumle, *Stem Cell Research: Toward Greater Unity in Europe?*, 139 Cell 649, 649–650 (2009).

100 International Society for Stem Cell Research, *Guidelines for Stem Cell Research and Clinical Translation*, 12 (2025). The latest version (V1.2 in 2025), however, calls for special oversight to weigh the extension of 14 days with the scientific objectives of the study based on necessity.

101 NGSCR, 2017, guideline 8.3.2.

102 Harshal Deshpande and others, *Stem Cell Banking: An Update on Current Scenario*, 3 Indian Journal of Research in Pharmacy and Biotechnology 392, 392 (2015).

103 NGSCR, 2017, guideline 14; Lahiry et al., *supra* note 55, 150.

104 Bindu S Perappadan, *ICMR bans banking of many stem cell sources*, The Hindu (14 Oct. 2017), <https://www.thehindu.com/news/cities/Delhi/icmr-bans-banking-of-many-stem-cellsources/article1986366-8.ece#:~:text=In%20its%20recommendation%2C%20ICMR%20has,not%20permitted%20until%20further%20notification>.

105 Drugs and Cosmetics (3rd Amendment) Rules, 2011.

106 Drugs Rules, 1945, rule 122F.

107
*Id*.

108
*See* NGSCR, 2017, guideline 14.1.2. However*,* in the case of HSCT, it is allowed under the respective guidelines (*See* HSCT Guidelines, 2021, guideline 6.3)*.*

109 Anupam Sachdeva and others, *Umbilical Cord Blood Banking: Consensus Statement of the Indian Academy of Pediatrics*, 55 Indian Pediatrics 489, 492 (2018).

110 Dhanya Lakshmi Narayan and Shubha R Phadke, *Concepts, Utility and Limitations of Cord Blood Banking: What Clinicians Need to Know*, 86 The Indian Journal of Paediatrics 44, 45 (2019).

111 Guidelines for Umbilical Cord Blood Banking Collection, Processing, Testing, Storage, Banking and Release for Clinical Application, 2023.

112 Kapoor, *supra* note 10.

113 Tiwari & Desai, *supra* note 17, 651.

114
*Id*., 650.

115 Drugs and Magic Remedies (Objectional Advertisements) Act, 1954, No.21, Acts of Parliament, 1954 (‘DMR Act, 1954’).

116 Indian Medical Council (Professional Conduct, Etiquette and Ethics) Regulations 2002, regulations 6.1, 7.22 (‘IMC Regulations’).

117 Drugs Rules, 1945, schedule J read with rule 106.

118
*See also* Tiwari & Desai, *supra* note 17, 651.

119 DMR Act, 1954; Drugs Rules, 1945, rule 106 read with Schedule J.

120 Guidelines for Umbilical Cord Blood Banking Collection, Processing, Testing, Storage, Banking and Release for Clinical Application, 2023, guideline 9.4.

121 IMC Regulations, 2002, regulation 7.1 read with chapter 8.

122
*Id*., chapter 8.

123 CPA, 2019, section 17.

124 Taylor Brinsfield, Noah Pinson & Aaron Levine, *The Evolution and Ongoing Challenge of Unproven Cell-Based Interventions*, 13 Stem Cells Translational Medicine 13, 13 (2024).

125 Tiwari & Raman, *supra* note 18, 429–430; Prasanna Kumar Patra and Margaret Sleeboom-Faulkner, *Bionetworking: Between Guidelines and Practice in Stem Cell Therapy Enterprise in India*, 7 Scripted 295 (2010).

126 Zubin Master et al., *Stem Cell Tourism and Public Education: The Missing Elements*, 15 Cell Stem Cell 267, 267 (2014).

127 Nishakanthi Gopalan, Siti Nurani Mohd Nor & Mohd Salim Mohamed, *Global Human Embryonic Stem Cell Laws and Policies and Their Influence on Stem Cell Tourism*, 37 Biotechnology Law Report 255, 260 (2018).

128 Samantha Lyons, Shival Salgaonkar, and Gerard Flaherty, *International Stem Cell Tourism: A Critical Literature Review and Evidence-Based Recommendations*, 14 International Health 132, 137 (2022).

129 Kathleen C. Laird, *Your Body, Your Cells? Direct-to-Consumer Marketing of Autologous Stem Cell Therapies in the United States, Japan, and Australia*, 55 Vanderbilt Journal of Transnational Law 139, 150 (2022).

130 Salter, *supra* note 25, 85, 86.

131 Eliza Barclay, *Stem-Cell Experts Raise Concerns about Medical Tourism*, 373 The Lancet – World Report 883, 883 (2009).

132 Iain Murray et al., *Rogue Stem Cell Clinics*, 102 The Bone & Joint Journal 148, 148 (2020).

133 Victor Fleishcer, *Regulatory Arbitrage*, 89 Texas Law Review 227, 229 (2010).

134 NGSCR, 2017; NEGBHR, 2017.

135
*See infra* the discussion on unenforceability of guidelines in ‘A. The lack of legislative recognition for critical guidelines’ and ‘B. The systemic procedural ambiguity in the approval and oversight of clinical trials’ in part II.

136 Lhendup Bhutia, *Hardcell*, Open (6 Dec. 2017), <https://openthemagazine.com/health/hardcell/#google_vignette> (accessed Oct. 23, 2025).

137 Shashank Tiwari, Paul Martin & Sujatha Raman, *Governing the Stem Cell Sector in India*, in Phuc Van Pham & Achim Rosemann (eds), Safety, Ethics & Regulations 254 (2017).

138
*Id*.

139 In the following discussion, I propose certain reforms to address the issues, including the issue of stem cell tourism in India. However, addressing unregulated stem cell tourism as a global phenomenon is a more ambitious task that requires equal attention but a task that falls beyond the scope of the current inquiry (*See* Lesley DeRenzo, *Stem Cell Tourism: The Challenge and Promise of International Regulation of Embryonic Stem-Cell Based Therapies*, 43 Case Western Reserve Journal of International Law 877 (2011).

140 Tamra Lysaght et al., *The Deadly Business of an Unregulated Global Stem Cell Industry*, 43 Journal of Medical Ethics 744, 745 (2017).

141 The ramifications of lax implementation of clinical trial rules and regulations on the vulnerable sections of the population attained attention in the 2010s. While the system conceived better safeguards in response to this necessity, it is high time that the Government conduct a regulatory audit to assess the effectiveness of the regulatory measures and update the existing practices in line with global standards like the Declaration of Helsinki (*also see* Arpita Sengupta, *Clinical Trials in India: A Way towards Impoverishment?*, 11 NALSAR Student Law Review 15, 21 (2017); Krishna Sarma and Manisha Singh, *Clinical Trials in India*, 2018 FDLI Update 32, 33, 34 (2018)).

142 Lyons, Salgaonkar, and Flaherty, *supra* note 128, 1.

143 Drugs Rules, 1945, rule 122B(2).

144 CT Rules, 2019, rule 25(vi).

145 Hurwitz, *supra* note 57.

146 Tiwari & Desai, *supra* note 17, 649; *also see* Question Nos. 2 and 3 in Appendix.

147 Bhutia, *supra* note 136.

148 Caroline Mathen, Neeta Devi, and Sinnapah Kang, *Stem Cell Research and Therapy in India: General Awareness for the Public and Stem Cell Therapy Providers*, 7 CHRISMED Journal of Health and Research 90, 90 (2020).

149 Tiwari & Desai, *supra* note 17, 649.

150 Dinesh Boopalan, Ramanan Pandian, and Gokul Kesavan, *Prospects for Stem Cell-Based Regenerative Therapies in India*, 3 StemJournal 11, 17 (2021).

151 Dalip Kaur v Union of India 2023 SCC OnLine Del 5491; *See* also Prashant Jha, *Delhi High Court Allows Continuation of Stem Cell Treatment for Two Children with Autism*, Bar and Bench (3 Sept. 2023), <https://www.barandbench.com/news/delhi-high-court-allows-stem-cell-treatment-continue-two-children-autism> (accessed Oct. 23, 2025).

152 id ¶6. The judgement reads, ‘*To date, neither the National Medical Commission nor the Government of India has issued a definitive order prohibiting stem cell therapy for ASD. Furthermore, no statutory provision has been presented before us explicitly forbidding the use of stem cell therapy in cases of ASD’.*

153 ICMR, *supra* note 79; *also see*  Table 1.

154 Angela Campbell & Kathleen Cranley Glass, *The Legal Status of Clinical and Ethics Policies, Codes, and Guidelines in Medical Practice and Research*, 46 McGill Law Journal 473, 485 (2001).

155 Prasanna Kumar Patra & Margaret Sleebom-Faulkner, *Bionetworking and Strategic Linking between India and Japan: How Clinical Stem Cell Intervention Continues despite New Regulatory Guidelines*, 11 East Asian Science, Technology and Society 353, 370 (2017).

156 NGSCR, 2017, guideline 11.2.

157
*Id*., Annexure 1.

158 The under-regulation of clinical trials was brought to light through a public interest litigation initiated by an NGO in Swasthya Adhikar Manch, Indore & Anr. v. Union of India & Ors*.,* Writ Petition (Civil) No. 33 of 2012 (Supreme Court of India).

159 17th LS Debates 2 March 2020, vol VII, no 10, page 41.

160 Jose, George and Thomas, *supra* note 18, 497.

161 Tiwari & Desai, *supra* note 17, 649.

162
*Id*.

163
*Id*., 650.

164 It is also true that these players may get over the advertisement laws by displaying incomplete information and clever description of data, thus giving an appearance of legitimacy. *See* Kandhari, *supra* note 20, 358; Casimir MacGregor, Alan Petersen & Megan Munsie, *From the Margins to Mainstream: How Providers of Autologous Stem Cell Treatments’ Legitimise Their Practice in Australia*, 25 Health 51, 60 (2021).

165 Nutech MediWorld, *Treatments*, <https://www.nutechmediworld.in/our-specialities/> (accessed Oct. 4, 2025).

166
*Shikha v. Union of India*, 2019 SCC OnLine Del 8856; *Deepak Khosla v. Union of India*, 2019 SCC OnLine Del 8857; *Shivam Singh v. Union of India*, 2019 SCC OnLine Del 8859; *Rita Devi v. Union of India*, 2019 SCC OnLine Del 8861; *Rahul Kaushik v. Union of India*, 2019 SCC OnLine Del 8862; *Raktim Debnath v. Union of India*, 2019 SCC OnLine Del 8864 (In all of these cases, the Delhi HC had allowed the petitioners/patients suffering from serios diseases to continue their stem cell therapy from Nutech MediWorld though it did not have license under the CT Rules. The Court allowed the treatments to continue on an interim basis as it was noted that abruptly ending the treatment would be harmful to their health).

167 Patra and Faulkner, *supra* note 125, 302.

168 Tiwari and Raman, *supra* note 18, 421; Kapoor, *supra* note 10.

169 CDSCO, *Introduction*, <https://cdsco.gov.in/opencms/opencms/en/About-us/Introduction/> (accessed Jan. 5, 2025).

170 Sandhya Srinivasan and Amar Jesani, *Standing Committee Report on CDSCO: Hard Facts Confirm and Open Secret*, 9 Indian Journal of Medical Ethics 148, 148–149 (2012).

171 Department Related Parliamentary Standing Committee on Health and Family Welfare, *The Functioning of the Central Drugs Control Standard Organization* (59th Report, 2012).

172 Agnihotri & Chandrashekaran, *supra* note 30, 14.

173
*Dalip Kaur v. Union of India* 2023 SCC OnLine Del 5491 ¶6.

174
*Shikha v. Union of India*, 2019 SCC OnLine Del 8856; *Deepak Khosla v. Union of India*, 2019 SCC OnLine Del 8857; *Shivam Singh v. Union of India*, 2019 SCC OnLine Del 8859; *Rita Devi v. Union of India*, 2019 SCC OnLine Del 8861; *Rahul Kaushik v. Union of India*, 2019 SCC OnLine Del 8862; *Raktim Debnath v. Union of India*, 2019 SCC OnLine Del 8864; *Abhishek Agarwal & Ors. v. Union of India & Anr. LNINDORD* 2019 Del 3104 ¶5.

175 Prasad, *supra* note 19, 745.

176 These have been spelt out in the next sub-section, Section IV.A.2.

177
International Bioethics Committee, The use of embryonic stem cells in therapeutic research, ¶¶53–4 (2001), <https://unesdoc.unesco.org/ark:/48223/pf0000132287> (accessed Oct. 23, 2025).

178 Sydney Hope, *When Miracle Cures Go Bad: Regulators’ Responses to Unproven Direct-to-Consumer Stem Cell Therapies Direct-to-Consumer Stem Cell Therapies*, 23 Science & Technology Law Review 257, 265 (2020).

179
*Id*., 271–273; Dana Jaalouk et al., *Regulatory Aspects of Regenerative Medicine in the United States and Abroad*, 5 Dermatological Review 1, 7 (2024).

180 Shreya Shrivastava & Dhvani Mehta, *Drug Regulation and Federalism in India*, in Balveer Arora & Mohan Rao (eds), Public Health and Federalism in India (2025).

181 Human Tissue Act, 2004 (United Kingdom); Human Embryology and Fertilisation Act, 2008 (United Kingdom).

182 Act on the Safety of Regenerative Medicine, 2014 (Japan).

183 For example, *see* Kayo Takashima, Michael Morrison & Jusaku Minari, *Reflection on the Enactment and Impact of Safety Laws for Regenerative Medicine in Japan*, 16 Stem Cell Reports 1425 (2021).

184
*See* Margaret Sleeboom-Faulkner, Haidan Chen & Achim Rosemann, *Regulatory Capacity Building and the Governance of Clinical Stem Cell Research in China*, 45 Science and Public Policy 416, 425 (2017); Prasad, *supra* note 19, 745.

185 IND CONST., sch 7 item number 6.

186 Chowdhary et al., *supra* note 31.

187 For example, *see* classification of cell-based therapies laid down by the European Medical Agency, *Commission Directive (EU) 2009/120/EC of 14 September 2009 Amending Directive 2001/83/EC of the European Parliament and of the Council on the Community Code Relating to Medicinal Products for Human Use as Regards Advanced Therapy Medicinal Products* [2009] OJ L242/3.

188 Geoffrey P. Lomax, Art Torres & Maria T. Millan, *Regulated, Reliable, and Reputable: Protect Patients with Uniform Standards for Stem Cell Treatments*, 9 Stem Cells Transnational Medicine 547, 550 (2020).

189 There is an argument that informed consent, no matter what, is not possible at all in cases of untested stem cell treatments, thus advocating for strict intervention from the State. *See* Laura Langford & Patrick Foong, *Unproven Stem Cell Therapies: An Evaluation of Patients’ Capacity to Give Informed Consent*, 33 Griffith Law Review 58, 84 (2024).

190 As pointed out by an empirical study in Australia, oftentimes clinics commercially sell therapies as ‘self-funded clinical trials’—that is, by making the patients bear the costs. This confounds the basic principle of a clinical trial. *See* Claire Tanner, Alan Petersen & Megan Munsie, *‘No one here’s helping me, what do you do?’: Addressing Patient Need for Support and Advice about Stem Cell Treatments*, 12 Regenerative Medicine 791, 797 (2017).

191 For a similar mechanism, *see* Motor Vehicles Act, 1988, No. 59, section 164A, Acts of Parliament, 1988, where an interim relief is granted by the Court by virtue of injury of death without going into the merits of the claim for immediate succor to the aggrieved person.

192 See the next sub-section on institutions. *Also see* Chowdhary et al., *supra* note 31, 30–31.

193 Leigh Turner, *US Stem Cell Clinics, Patient Safety, and the FDA*, 21 Trends in Molecular Medicine 271, 272. (2015).

194 More on this is discussed in detail in a later sub-section on establishing dedicated funding mechanism.

195 Lomax, Torres & Millan, *supra* note 188.

196 The Medicines for Human Use (Clinical Trials) Regulations 2004, reg 49 (UK); Shaun d Pattinson, Medical Law and Ethics, 397–398 (2014).

197
*See* 14th LS Debates 24 August 2005, vol XIII, no 21, pages 2, 371 (here, the potential of stem cell research is appreciated by the members and calls for further funding of these studies); 14th LS Debates 10 December 2008, vol XXXV, no 9, page 765 (here, the government acknowledges that the illegal use of stem cells has come to its notice—but no record of such cases have been maintained); 17th LS Debates 2 March 2020, vol VII, no 10, page 41 (here, a member raised concerns regarding the negative impact on stem cell research due to the unsystematic policies of ICMR).

198 David Barnhizer, *What Causes Law to Succeed or Fail?*, Cleveland-Marshall Legal Studies Paper No. 297, 5–6 (2016), <https://papers.ssrn.com/sol3/papers.cfm?abstract_id=2744885#:~:text=David%20Barnhizer-Cleveland%2DMarshall%20College&text = There%20is%20a%20ruthless%20power,good%20intentions%E2%80%9D%20or%20pipe%20dreams >, (accessed Oct. 4, 2025).

199 Bin Wei, Shuyao Cheng & Yang Feng, *Neural Personal Information and Its Legal Protection: Evidence from China*, 12 Journal of Law and Biosciences 1, 15 (2025); Emma Cave, *Advocating Distinct Regulatory Paths for Embryos and Embryo-Like Structures*, 12 Journal of Law and Biosciences 1, 22 (2025).

200
*See infra* ‘IV.C Establishing a dedicated funding mechanism’.

201 Mihika Poddar & Bhavya Nahar, *‘Continuing Mandamus’—A Judicial Innovation to Bridge the Right-Remedy Gap*, 10 NUJS L.R. 555, 568 (2017).

202 Vaishnav M, *A Tale of Two Friends: How Performance Audit Complements Judicial Review*, The Leaflet (27 Sept. 2024), <https://theleaflet.in/explainer/a-tale-of-two-friends-how-performance-audit-complements-judicial-review> (accessed Oct. 23, 2025).

203 Barnhizer, *supra* note 198, 7.

204 59th Report, *supra* note 171; *also see* Srinivasan & Jesani, *supra* note 170.

205 Agnihotri & Chandrashekaran, *supra* note 30.

206 D&C Act, 1940, s 7.

207 Chowdhary, *supra* note 31, 7.

208
*Id*. 23.

209 Turner, *supra* note 193, 271.

210 Chowdhary, *supra* note 31, 30–31.

211 Tiwari, Martin & Raman, *supra* note 137, 255.

212 The State MCs often come to know about such instances of violation only when brought to its attention via complaints. For example, see Lata Mishra, *Mumbai: Cheat Radiologist Who Gave Multi-Vitamins in the Name of Stem Cell Therapy Loses Licence*, Mumbai Mirror (7 Aug. 2018), https://mumbaimirror.indiatimes.com-/mumbai/cover-story/cheat-radiologist-who-gave-multi-vitamins-in-the-name-of-stem-cell-therapy-loses-licence/articleshow/65299429.html .

213 CDSCO reforms have already been suggested through Mashelkar Committee and the 59th Parliamentary Report, *supra* note 171. Several scholars have also similarly suggested—*see* Agnihotri and Chandrasekharan, *supra* note 30; Chowdhary, *supra* note 31.

214
Steve Olson & Stephen Merrill (eds), Measuring the Impacts of Federal Investments in Research 172 (2011).

215 Press Information Bureau, *Research in Regenerative Medicine* (11 Feb. 2022), available at <https://pib.gov.in/PressReleaseIframePage.aspx?PRID=1797695> (accessed Oct. 4, 2025).

216
*Id.*

217
*Id*; *also see* Question 6 in Appendix that reveals how the ICMR funding has fluctuated over the years.

218
*Id*.

219 Salter, *supra* note 25, 152

220 Lahiry et al., *supra* note 55, 149.

221
*Fund Sought for Stem Cell Research*, Business Standard (5 Apr. 2005), <https://www.business-standard.com/article/economy-policy/fund-sought-for-stem-cell-research-105040501079_1.html> (accessed Oct. 23, 2025).

222 European Commission, *Horizon Europe*, <https://research-and-innovation.ec.europa.eu/funding/funding-opportunities/funding-programmes-and-open-calls/horizon-europe_en> (accessed Mar. 31, 2025).

223
*See* Article 18 *in* European Council Regulation (EU) 2021/695 of the European Parliament and of the Council of 28 April 2021 establishing Horizon Europe—the Framework Programme for Research and Innovation, laying its rules for participation and dissemination, and repealing Regulations (EU) No 1290/2013 and (EU) No 1291/2013 <https://eur-lex.europa.eu/eli/reg/2021/695/oj> (accessed Mar. 29, 2025).

224
*Id*., Article 19.

225 Pandey and Desai, *supra* note 15.

226
Vijay Kelkar & Ajay Shah, In Service of the Republic: The Art & Science of Economic Policy, 53 (2019).

227 Amit Prasad, *Biopolitical Excess: Techno-Legal Assemblage of Stem Cell Research in India*, 22 Science, Technology and Society 102, 115 (2017).

228 Kandhari, *supra* note 20.

229
*Id*.

230
*Id* 8.

231 Video posts have been found to be highly persuasive [*See* Bethany Hawke et al., *How to Peddle Hope: An Analysis of YouTube Patient Testimonials of Unproven Stem Cell Treatments*, 12 Stem Cell Reports 1186 (2019); Robin Downey and Rose Geransar, *Stem Cell Research, Publics' and Stakeholder Views,* 16 Health L Rev 69, 70 (2008)].

232 The said studies were based on a survey conducted in Saudi Arabia, another developing country (*See* Doaa Aboalola et al., *An Infodemic of Misinformation on Stem Cell Therapy Among the Population of Saudi Arabia: A Cross-Sectional Study*, 9 Frontiers in Medicine (2022), <https://www.frontiersin.org/articles/10.3389/fmed.2022.789695/full> (accessed Mar. 31, 2025); Doaa Aboalala et al., *Public Awareness and Understanding of Stem Cell Treatments Available in Saudi Arabia and Their Trust in Hospitals and Research Centres Involved in Stem Cell Research—A Cross Sectional Study*, 12 Frontiers in Public Health (2024), <https://www.frontiersin.org/articles/10.3389/fpubh.2024.1364809/full> (accessed Mar. 31, 2025).

233 Tanner, Petersen & Munsie, *supra* note 190, 798.

234
*See* Question No 5 of Appendix 1.

235 Considering information asymmetry, the consumers find it difficult to acknowledge the risks and ambiguities associated with the unproven therapies—especially when they are promoted by well-known experts in the medical field (*For example, see* David Fitzpatrick and Drew Griffin, *Family Hangs Hope for Boy on Unproven Therapy in India*, CNN (21 May 2021), <https://edition.cnn.com/2012/05/19/health/embryonic-stem-cell-therapy/index.html> (accessed Mar. 31, 2025)*; Also see* Kandhari, *supra* note 20, 8.

236 Alan C Regenberg, *Stem Cell Science Should Be Tweeted*, 9 Regenerative Medicine 125 (2014).

237 Sengupta, *supra* note 141, 19.

238 Kayo Takashima, Michael Morrison & Jusaku Minari, *Reflection on the Enactment and Impact of Safety Laws for Regenerative Medicine in Japan*, 16 Stem Cell Reports 1425, 1430 (2021).

239 Prasad, *supra* note 19, 744; Tanner, Petersen & Munsie, *supra* note 190, 797.

240 Insoo Hyun, *Therapeutic Hope, Spiritual Distress, and the Problems of Stem Cell Tourism*, 12 Cell Stem Cell 505, 505–506 (2013).

241
*Id*. 506.

242
*Id*., 507; Joe Hannan, *Working with Patients who Want Unproven Treatments*, MD Linx (4 May 2022), <https://www.mdlinx.com/article/working-with-patients-who-want-unproven-treatments/2LVndv1n09aWSgAs77K4Gp> (accessed Oct. 4, 2025).

243 Heitmeyer, *supra* note 20, 209.

244 This assistance that the patient group can lend in civil, criminal or consumer dispute litigation would be helpful in resolving the problem question—‘who notices if something goes wrong with respect to medical practitioner’s conduct or service’, as posited in Tiwari, Martin & Raman, *supra* note 137, 256.

245 Heitmeyer, 20.

246 This suggestion applies to unproven therapies generally, not just stem cell therapy.

247 In the current framework, stem cell research participation has been limited to only a few premier institutions, leading to a situation of disparity [*See* S. Gopalakrishnan et al., *Scientometric Analysis of Stem Cell Research: A Comparative Study of India and Other Countries*, 6 Collnet Journal of Scientometrics and Information Management 229, 248 (2012)].

248 Pranbihanga Borpuzari, *Advancing Stem Cell Therapy: The Regulatory Process to Get to Clinical Trials*, The Economic Times (4 Mar. 2024), <https://economictimes.indiatimes.com/small-biz/entrepreneurship/advancing-stem-cell-therapy-the-regulatory-process-to-get-to-clinicaltrials/articleshow/-108193130.cms?from=mdr> (accessed Oct. 23, 2025).

## Appendix A: RTI Queries and Replies

1. How many licensed Institutional Committees for Stem Cell Research (IC-SCRs) are currently operating in India, and can you provide a list of their locations and areas of specialization?
  Reply: All ICSCRs have been dissolved as per the OM issued by DHR, MoHFW, dated 3 March 2024.
2. What is the nature and scope of actions that can be taken against those who violate stem cell research and therapy regulations? Kindly provide instances of enforcement actions taken against violations of stem cell research regulations during the period 2019–2024.
  Reply: ICMR is not an enforcement agency.
3. How does the regulatory body evaluate the safety and efficacy of investigational stem cell therapies, prior to the approval for clinical trials? Are there established tests that are to be compulsorily conducted to evaluate the safety of such stem cell therapies?
  Reply: ICMR is not a regulatory body.
4. What are the mechanisms in place to identify unauthorized stem cell research and therapies? How many unauthorized stem cell research and therapies were identified during the period 2010–2024?
  Reply: ICMR does not monitor such activities as its mandate is to conduct medical research.
5. What are the initiatives to educate the public about the risks and benefits of stem cell therapies? What are the initiatives to make hospitals, clinics, and laboratories aware of regulatory compliance requirements?
  Reply: Therapy is not under the purview of ICMR. However, to bring clarity to the stakeholders, ICMR, in collaboration with DBT, has issued the following guidelines and the same are available in public domain on the following link:

- National Guidelines Stem Cell Research (https://dbtindia.gov.in/sites/default/files/National_Guidelines_StemCellResearch-2017.pdf).
- Evidence-Based Status of Stem Cell therapy in Human Diseases (https://main.icmr.nic.in/sites/default/files/upload_documents/Evidence_Based_Status_of_Stem_Cell_Therapy_for_Human_Diseases_v1.pdf).

1. Which are the Government Departments/Bodies/Agencies that fund stem cell research in India? Is there any specific strategy/plan/scheme to fund stem cell research in India? Kindly provide the details of funds allocated by each government funding institutions/bodies/agencies for stem cell research in the past five years.
  Reply: ICMR being the apex body in India for the formulation, coordination, and promotion of biomedical research has supported research projects under the area of stem cell research through extramural research program every year. Annexure on details of fund for stem cell research in the last five years is attached (See Table 2).

**Table 2:** Funding trends of ICMR over the years

**Year**	**Total funds released (Rs.)**
2019	4,36,19,531/−
2020	68,82,564/−
2021	2,17,72,579/−
2022	51,94,884/−
2023	62,71,655/−

